# Downregulation of the Werner syndrome protein induces a metabolic shift that compromises redox homeostasis and limits proliferation of cancer cells

**DOI:** 10.1111/acel.12181

**Published:** 2013-12-01

**Authors:** Baomin Li, Juan Manuel Iglesias-Pedraz, Leng-Ying Chen, Fei Yin, Enrique Cadenas, Sita Reddy, Lucio Comai

**Affiliations:** 1 Department of Molecular Microbiology and Immunology, Keck School of Medicine, University of Southern CaliforniaLos Angeles, CA, 90089, USA; 2 Institute for Genetic Medicine, Keck School of Medicine, University of Southern CaliforniaLos Angeles, CA, 90089, USA; 3 Department of Pharmacology and Pharmaceutical Sciences, School of Pharmacy, University of Southern CaliforniaLos Angeles, CA, 90089, USA; 4 Department of Biochemistry and Molecular Biology, Keck School of Medicine, University of Southern CaliforniaLos Angeles, CA, 90089, USA

**Keywords:** aging, DNA methylation, molecular biology of aging, muscle, epigenetic

## Abstract

The Werner syndrome protein (WRN) is a nuclear protein required for cell growth and proliferation. Loss-of-function mutations in the Werner syndrome gene are associated with the premature onset of age-related diseases. How loss of WRN limits cell proliferation and induces replicative senescence is poorly understood. Here, we show that WRN depletion leads to a striking metabolic shift that coordinately weakens the pathways that generate reducing equivalents for detoxification of reactive oxygen species and increases mitochondrial respiration. In cancer cells, this metabolic shift counteracts the Warburg effect, a defining characteristic of many malignant cells, resulting in altered redox balance and accumulation of oxidative DNA damage that inhibits cell proliferation and induces a senescence-like phenotype. Consistent with these findings, supplementation with antioxidant rescues at least in part cell proliferation and decreases senescence in WRN-knockdown cancer cells. These results demonstrate that WRN plays a critical role in cancer cell proliferation by contributing to the Warburg effect and preventing metabolic stress.

## Introduction

Werner syndrome (WS) is an autosomal recessive premature aging disorder that predisposes to the early onset of diseases observed during normal aging such as arteriosclerosis, osteoporosis, and type II diabetes mellitus (Epstein *et al*., 1966). Werner syndrome is caused by mutations in the gene that encodes a protein named Werner syndrome protein (WRN) with helicase and exonuclease activity (Yu *et al*., 1996). Biochemical studies have implicated WRN in cellular pathways that monitor and maintain DNA integrity (Rossi *et al*., 2010), although its precise function in these processes is not understood. Fibroblasts from WS individuals have a short replicative lifespan and display genomic instability, which manifests with an increased rate of chromosomal translocation and deletions (Salk *et al*., 1985; Fukuchi *et al*., 1989). *Wrn*^*−/−*^ mice do not show signs of premature aging, but develop symptoms of metabolic dysfunction when fed a high-fat diet (Lombard *et al*., 2000; Moore *et al*., 2008). Mice lacking WRN helicase activity (*Wrn hel*^*−/−*^), on the other hand, display many of the WS phenotypes and exhibit genomic instability and oxidative DNA damage (Massip *et al*., 2006). The underlying causes of oxidative stress, which has also been observed in patients with WS (Pagano *et al*., 2005), are unknown.

Patients with WS exhibit susceptibility to rare forms of cancer, primarily of mesenchymal origin (Goto *et al*., 1996), but do not show a significant increase in the incidence of epithelial tumors, which are the most common type of cancers in the general population. Interestingly, many cancer cell lines express relatively high levels of WRN, which is necessary for growth, anchorage-independent growth, and tumor formation in animal models (Opresko *et al*., 2007; Futami *et al*., 2008; Arai *et al*., 2011). Consistent with these findings, WRN is necessary for BCR/ABL and Myc-induced oncogenesis (Slupianek *et al*., 2011; Moser *et al*., 2012). Collectively, these data indicated that WRN is required for the proliferation of most cancer cells. To gain insights into the role that WRN plays in cell proliferation, we carried out proteome studies to identify the early cellular changes induced by WRN depletion. This analysis reveals an unanticipated link between WRN and metabolic pathways that regulate macromolecular synthesis, energy production, and redox balance. Importantly, we show that the metabolic shift induced by WRN depletion markedly increases the accumulation of reactive oxygen species (ROS) in cancer cells and contributes to the increased levels of oxidative DNA damage in these cells. These results demonstrate that depletion of WRN effectively inhibits tumor cell proliferation by inducing a metabolic shift that results in oxidative stress.

## Results

### Acute depletion of the WRN alters the levels of metabolic enzymes involved in energy production, macromolecule synthesis, and redox control

**Figure 1:**
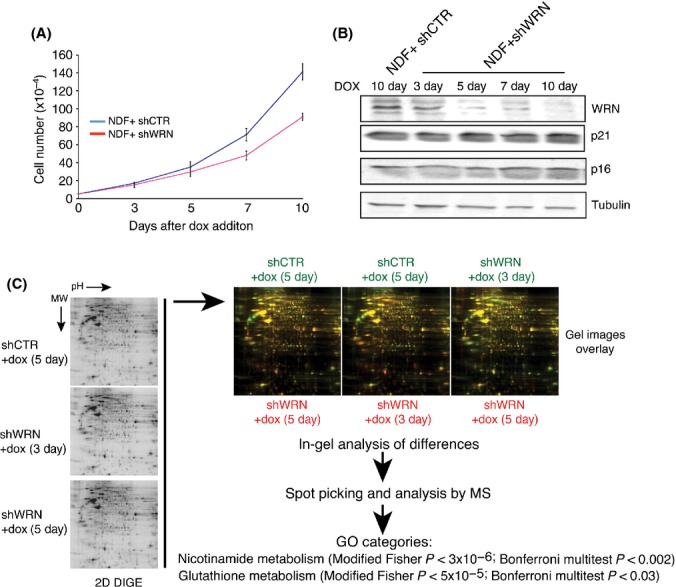
Werner syndrome protein (WRN) depletion affects the pathways linked to nicotinamide and glutathione metabolism. (A) Growth curves of NDF expressing shRNAs for WRN or green fluorescence protein (GFP). One microgram per millilitre doxycycline was added to the media, and the growth rate of each line was measured by counting viable cells at the indicated days. Cells were seeded at a low density, and the medium was changed every 2 days. Values represent the mean ± the standard deviation of three experiments (*n* = 3). (B) Western blot analysis showing the levels of WRN, p21, and p16 in normal diploid human fibroblasts (NDF) transduced with lentiviral vector for the conditional expression of shRNA targeting GFP (shCTR) and WRN (shWRN) at different days after the addition of doxycycline (1 μg mL^−1^). α-Tubulin was used as loading control. (C) 2D differential in-gel electrophoresis of extracts prepared from control fibroblasts expressing shCTR for 5 days and fibroblasts expressing shWRN for 3 and 5 days. Gene ontology analysis of pathways enriched in proteins that are affected in WRN-knockdown cells identifies nicotinamide and glutathione metabolism as the most significantly altered pathways in WRN-knockdown cells. Biological pathways that are significant (*P* < 0.05) using the Bonferroni multitest are shown.

To better understand the role of WRN in cell homoeostasis, we sought to define the early cellular changes that are triggered by the depletion of WRN. For this purpose, we generated microRNA-based short hairpins (shRNA-mirs) lentiviral vectors for the conditional knockdown of WRN in human cells. Upon induction with doxycycline (dox), expression of shRNAs targeting WRN (shWRN) results in the downregulation of WRN and a gradual reduction in the proliferation of human fibroblasts (Figs 1A,B and S1, Supporting Information). Using this system, we then compared the proteomes of cells expressing shWRN for 3 and 5 days with those of control cells expressing shRNAs against the green fluorescence protein (GFP) (shCTR) for 5 days by two-dimensional difference gel electrophoresis (2D DIGE) (Marouga *et al*., 2005) followed by comparative analysis of all spots using DeCyder differential in-gel analysis (DIA) software. Within the time frame of this analysis, the levels of p21 and p16, two major determinants of the senescence-inducing signals (Campisi & d’Adda di Fagagna, 2007), are not affected by WRN depletion (Fig. 1B). Protein spots whose signals were increased or decreased in intensity at both 3 and 5 days after shWRN induction when compared with shCTR were subjected to mass spectrometry (MS). This analysis identified 41 proteins whose signals were differentially expressed in the shWRN samples compared to shCTR, as shown by either red or green color in a two-way comparison (Figs 1C and S2A, Supporting Information). These proteins were clustered into functional groups using the Database for Annotation, Visualization, and Integrated Discovery (DAVID). This analysis revealed a remarkable enrichment in functional gene ontology (GO) groups involved in basic metabolic pathways, with the nicotinamide and glutathione metabolic processes being the most statistically significant (Figs 1C, S2B, and Table S1, Supporting Information). Two of the proteins within these pathways that were identified in our analysis, glucose 6 phosphate dehydrogenase (G6PD), the rate-limiting enzyme of the pentose phosphate pathway (PPP), and cytoplasmic nicotinamide adenine dinucleotide phosphate (NADP)-dependent isocitrate dehydrogenase (IDH1), are the major producers of cytoplasmic NADPH, a key provider of reducing equivalents for biosynthetic reactions and for protecting the cell against the toxicity of ROS. To assess the generality of this observation, we examined *Wrn*^*−/−*^ mouse embryonic fibroblasts (MEFs) and confirmed that G6PD and IDH1, as well as transketolase-like 1 (TKTL1), the enzyme controlling the nonoxidative part of the PPP whose levels were also affected by WRN knockdown in fibroblasts, are reduced in *Wrn*^*−/−*^ MEFs when compared to *WT* MEFs (Fig. S3, Supporting Information). For these experiments, MEFs were grown in low oxygen tension to prevent early replicative arrest (Parrinello *et al*., 2003).

**Figure 2:**
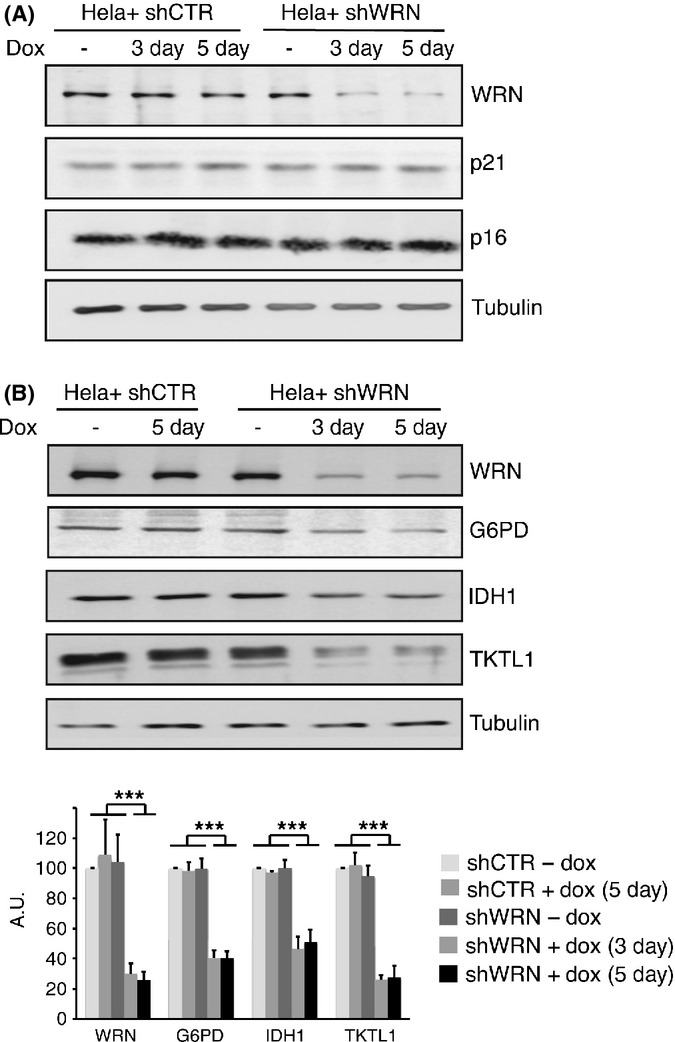
Werner syndrome protein (WRN) depletion alters the levels of metabolic enzymes. (A) Western blot analysis showing the abundance of WRN, p21, and p16 in control and shWRN cells before and after induction with doxycycline. α-tubulin was used as normalizing factor. (B) Representative western blots showing the levels of G6PD, IDH1, and TKTL1 in HeLa cells before and at 3 and 5 days after the induction of shRNAs against WRN or green fluorescence protein (shCTR). Quantification of chemiluminescent signals from western blots of three biological replicates was carried out using the Image Analyzer LAS-4000 (*Fujifilm* Life Science, Stamford, CT, USA) as described in the Data S1, and mean relative values [shCTR-dox set at 100 arbitrary units (a.u.)] ± SD are shown. α-Tubulin was used as normalizing factor. ***Denotes a *P* value <0.00001.

We reasoned that the metabolic shift induced by WRN knockdown would be expected to cause acute stress overload in cancer cells, but not in normal cells. This is because many cancer cells unlike normal cells exhibit a metabolic behavior, known as ‘Warburg effect’, that downregulates mitochondrial function and redirects glucose catabolism into the pentose phosphate pathway to sustain their increased requirements of proliferation through the generation of intermediates and reducing equivalents necessary for the *de novo* synthesis of nucleotides and for the control of ROS (Cairns *et al*., 2011). To test this idea, we transduced cervical cancer (Hela) and breast cancer (MCF7) cells with conditional shRNAs targeting WRN or GFP and we observed a rapid suppression of proliferation and an increase in the frequency of cells stained for senescence-associated β-galactosidase (SA-β-gal) after the induction with doxycycline (Fig. S4, Supporting Information), which is consistent with a previous study (Opresko *et al*., 2007). Neither p21 nor p16 levels were increased after WRN-knockdown Hela cells (Fig. 2A). However, downregulation of WRN was accompanied by an increase in the levels of p21 in MCF7 cells, a cancer cell line that displays a robust increase in the frequency of cells stained for SA-β-gal after WRN depletion (Fig. S5A, Supporting Information). To experimentally test whether the metabolic changes induced by WRN knockdown contribute to inhibiting the proliferation of cancer cells, we examined the levels of a representative set of key metabolic enzymes involved in the control of oxidative stress before and after WRN knockdown in Hela and MCF7 cells by western blotting. The results of this analysis demonstrated decreased levels of G6PD, TKTL1, and IDH1, in both cell lines at 3 and 5 days after the induction of shWRN (Figs 2B, S5B and S6, Supporting Information). Decreased levels of G6PD, TKTL1, and IDH1 at 3 and 5 days after the induction of shWRN were also observed in cells cultured in 1% serum (Fig. S7A, Supporting Information). To ensure that the altered protein levels were a consequence of WRN knockdown and not due to artifacts caused by the method utilized to silence WRN, these results were confirmed by the analyses of Hela cells transfected with siRNAs targeting WRN or scrambled siRNAs as control (Fig. S7B). We also asked whether oxygen levels influence these changes in metabolic enzymes. For this purpose, we incubated cancer cells in 1% oxygen, a growth condition that mimics that of most solid tumor microenvironment (Cairns *et al*., 2011), and observed a significant decrease in the levels of G6PD, TKTL1, and IDH1 in both Hela and MCF7 after WRN knockdown (Fig. S8, Supporting Information) that was comparable to that of cells grown under atmospheric oxygen. This result indicates that the metabolic alterations induced by WRN knockdown are independent of oxygen concentration.

### Downregulation of WRN affects antioxidant defenses and mitochondrial respiration, which leads to increased levels of ROS and oxidative DNA damage in cancer cells

**Figure 3:**
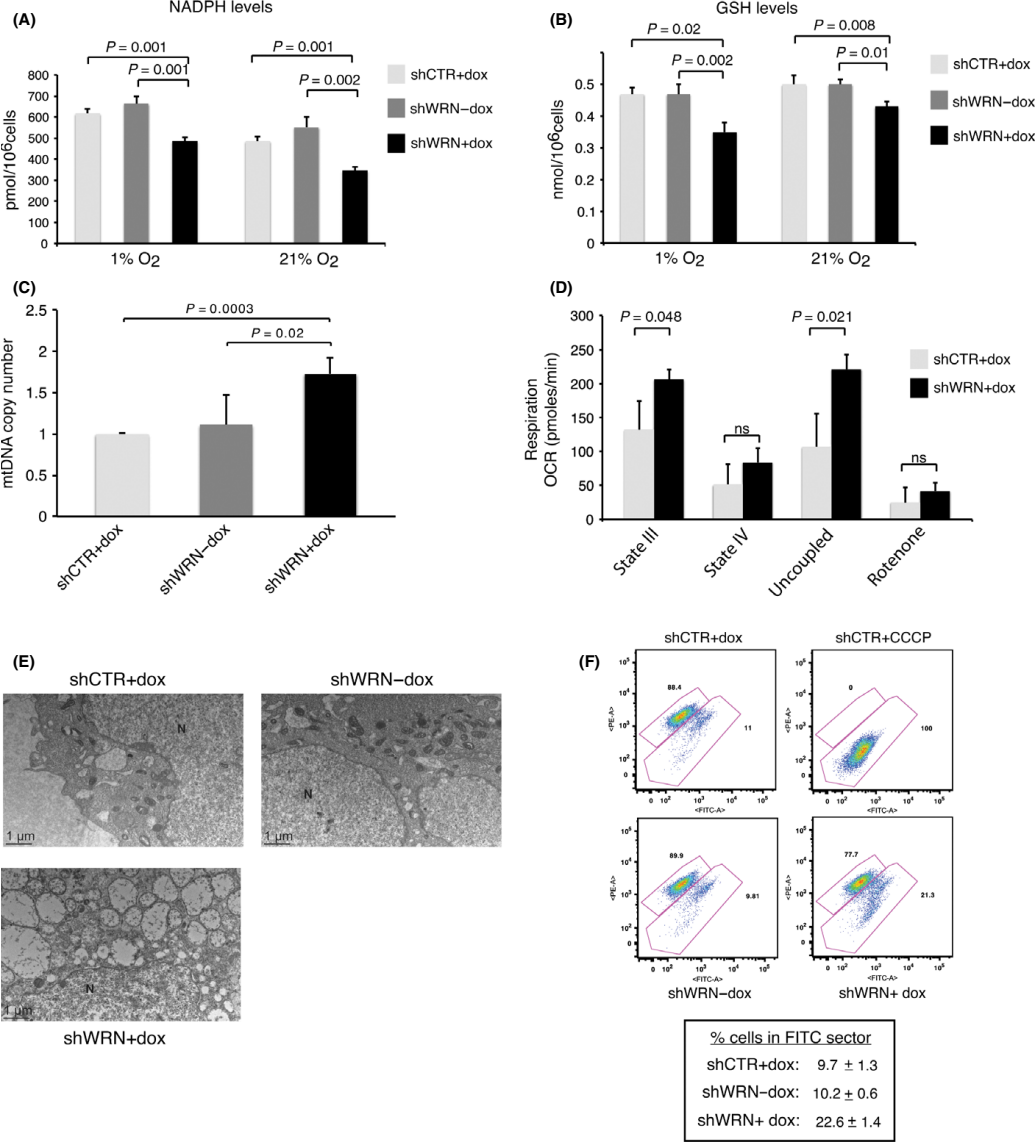
Changes in the levels of metabolic enzymes in Werner syndrome protein (WRN)-knockdown cancer cells result in reduced levels of NADPH and reduced glutathione (GSH) and altered mitochondrial function. (A) NADPH levels were measured in Hela cells transduced with lentiviral vectors for the expression of shRNAs against green fluorescence protein (GFP) or WRN that were grown in the presence of doxycycline (+dox) for 3 days under 21% or 1% oxygen. (B) GSH levels were measured in Hela cells transduced with lentiviral vectors for the expression of shRNAs against GFP or WRN that were grown in the absence or presence of doxycycline (+dox) for 3 days in an atmosphere of 21% or 1% oxygen. Each data point represents the mean ± SD of three biological replicates, and *P* values were calculated by two-tailed Student’s *t*-test. (C) Mitochondrial DNA quantification by qPCR in Hela cells expressing conditional shRNAs for WRN before and after the induction or GFP controls. Mean values (*n* = 4) ± SDs are shown, and *P* values were calculated by two-tailed Student’s *t*-test. (D) Oxygen consumption rates in HeLa cells transduced with lentiviral vectors for the expression of siRNAs against GFP or WRN that were grown in the absence or presence of doxycycline (+dox) for 5 days. Maximal respiratory capacity was measured after the addition of the uncoupler carbonylcyanide-*p*-trifluoromethoxyphenylhydrazone. Each data point represents the mean ± SDs of three independent experiments, each of which was carried out using five replicates. (E) Representative transmission electron microscopy images (2500×) of Hela cells for the inducible expression of shRNAs against WRN or GFP showing swollen mitochondria in Hela cells 3 days after WRN knockdown. N, nucleus. (F) Mitochondrial membrane potential in Hela cells transduced with lentiviral vectors for the expression of shRNAs against WRN or GFP grown in the absence (-dox) or in the presence of doxycycline (+dox) for 3 and 5 days. Cells were stained with 2 μm JC-1 for 15 min at 37 °C, 5% CO_2_ and then washed with phosphate-buffered saline and analyzed on a flow cytometer using 488-nm excitation with 530-nm and 585-nm bandpass emission filters. Cells were binned into two sectors of either red (high membrane potential) or green (low membrane potential) fluorescence. An increase in the percentage of cells with green fluorescence after WRN knockdown reflects a decrease in membrane potential. The uncoupler CCCP was added as a control for membrane depolarization: note the shift of cells to the green sector indicative of depolarized mitochondria. Representative FACS analysis and mean value ± SD of percentage of cells in FITC (green) sector from three biological replicates are shown.

Glucose 6 phosphate dehydrogenase and IDH1 are major producers of cytoplasmic NADPH, the coenzyme that provides reducing equivalent for the regeneration of cytosolic reduced glutathione (GSH). To experimentally test whether reduced abundance of G6PD and IDH1 negatively affected the cellular antioxidant system, we measured NADPH levels in WRN-knockdown Hela cells grown at either 21% or 1% oxygen and found that it was significantly lower than in the control cells (Fig. 3A). As NADPH is utilized by glutathione reductase to generate GSH for ROS detoxification, we then asked whether GSH levels are influenced by WRN knockdown. The results of this experiment show that Hela cells grown in either 1% or 21% O_2_, as well as cells grown in 1% serum, show reduced levels of GSH after WRN knockdown (Figs 3B and S9A, Supporting Information), demonstrating that WRN depletion influences the cellular antioxidant system and the ability of cancer cells to detoxify ROS.

**Figure 4:**
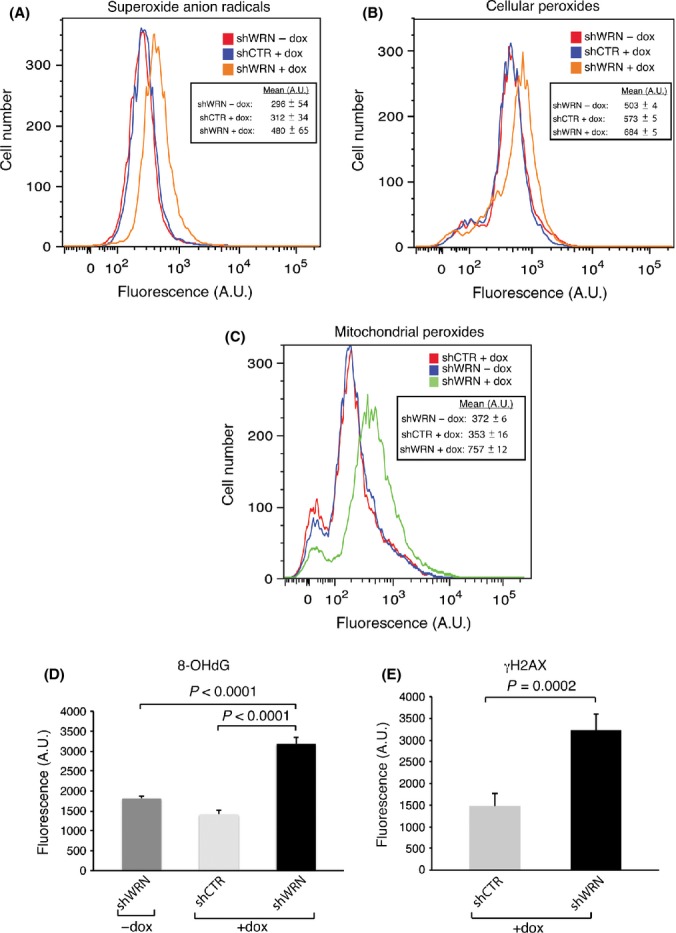
Reactive oxygen species accumulation and oxidative damage to DNA in Werner syndrome protein (WRN)-knockdown cancer cells. (A) Superoxide anion radicals generated by the mitochondria in Hela cells transduced with lentiviral vectors for the expression of siRNAs against green fluorescence protein (GFP) or WRN that were grown in the absence (−) or presence (+) of doxycycline (dox) for 5 days were determined using MitoSOX Red and analyzed by flow cytometry. Histogram from representative experiment and mean fluorescence emission for each sample from two independent replicates are shown. (B) Measurement of intracellular oxidative status after WRN knockdown. Experiments were performed exactly as in (A), and peroxide levels (primarily H_2_O_2_) were detected using the fluorescent indicator Peroxy Fluor-6 (PF-6) by flow cytometry, as described in Experimental procedures. Histogram from representative experiment and mean fluorescence emission for each sample from two independent experiments are shown. (C) Measurement of mitochondrial oxidative status in Hela cells transduced with lentiviruses for the conditional expression of shRNAs targeting WRN or GFP grown in the absence (−) or presence (+) of doxycycline (dox) for 5 days. Cells were incubated with mitochondrial Peroxy Yellow 1 (mitoPY-1) as described in Experimental procedures and subjected to analysis by flow cytometry. Histogram from representative experiment and mean emission intensities for each sample from three independent replicates are shown. (D) Hela cells transduced with lentiviral vectors for the expression of shRNAs against WRN or GFP (shCTR) were grown in the absence or presence of doxycycline (+dox), and the oxidized nucleoside 8 hydroxy-2′-deoxyguanosine was detected by immunofluorescence microscopy. Quantification of total nuclei fluorescence for each sample was carried out using Image J software (National Institute of Health, Bethesda, MD, USA). Mean values of two independent experiments (*n* > 100 nuclei per experiment) ± SDs are shown, and *P* values were calculated by two-tailed Student’s *t*-test. (E) Hela cells transduced with lentiviral vectors for the expression of shRNAs against WRN or GFP (shCTR) were grown in the absence or presence of doxycycline (+dox), and phosphorylated histone H2AX (γH2AX) was detected by immunofluorescence microscopy and signal was quantitated as described above.

In cancer cells, most of the glucose flux is diverted away from the mitochondria into the pentose phosphate pathway to generate reducing potential for ROS detoxification. These data prompted us to test whether changes in WRN levels affect the function of mitochondria in these cells. Quantitative polymerase chain reaction (qPCR) analysis showed an ~1.7-fold increase in mitochondrial DNA (mtDNA) content in WRN-knockdown Hela cells compared to controls (Fig. 3C). Analysis of mitochondrial respiration demonstrated increases in both basal and uncoupled [induced by carbonylcyanide-*p*-trifluoromethoxyphenylhydrazone (FCCP)] oxygen consumption (Figs 3D and S9B). Consistent with the qPCR data, we observed an increased number of mitochondria in WRN-depleted Hela cells by transmission electron microscopy (Fig. 3E). Remarkably, this analysis revealed that most mitochondria in the WRN-knockdown cells are swollen and display severely reduced matrix density. These morphological changes were accompanied by a decrease in mitochondrial membrane potential, as determined by the measurements of JC-1 fluorescence ratio by flow cytometry (Fig. 3F). Because these alterations are generally associated with oxidative stress (Vercesi *et al*., 1997; Seo *et al*., 2010), we then tested whether downregulation of WRN elicits the accumulation of ROS. We measured the intracellular concentration of superoxide ions using the fluorogenic dye Mitosox Red and demonstrated that downregulation of WRN leads to a significant increase in the levels of superoxides when compared to control cells (Fig. 4A). Superoxide anions generated in the mitochondria are converted into hydrogen peroxide, which can freely diffuse through the mitochondrial membrane and contribute to oxidative stress in the cytoplasm and nucleus. To determine whether increased levels of peroxides accompanied the increase in superoxide anions, we analyzed cells using Peroxy Fluor-6 (PF-6) and mitochondrial Peroxy Yellow-1 (mitoPY-1), two fluorescent probes that detect primarily hydrogen peroxide in the whole cell or specifically in the mitochondria, respectively (Dickinson & Chang, 2008; Dickinson *et al*., 2011). We observed a substantial increase in fluorescence with PF-6 and mitoPY-1 after WRN knockdown, demonstrating that global intracellular levels as well as mitochondria levels of peroxides are significantly higher in WRN-depleted cancer cells than in control cells (Fig. 4B,C). These results suggest that WRN plays an important role in cancer cells redox homeostasis by limiting the accumulation of ROS.

Cancer cells are critically dependent on a strong antioxidant system and are highly vulnerable to oxidative stress and damage to macromolecules including DNA (Trachootham *et al*., 2009). To test whether increased levels of ROS resulted in the accumulation of oxidative damaged products, we measured the levels of 8-hydroxy-2-deoxyguanosine (8-OHdG), one of the most common oxidative DNA lesions, and phosphorylated histone H2AX (γH2AX), a marker of DNA double-strand breaks, which can also arise following replication past oxidative DNA lesions, in WRN-knockdown and control Hela cells. These analyses demonstrated that WRN-knockdown cells exhibit higher levels of 8-OHdG and γH2AX (Figs 4D,E and S9C), indicating that reduced redox buffering capacity after downregulation of WRN leads to the accumulation of oxidative damage and DNA breaks. The oxidative stress induced by downregulation of WRN in Hela cells was not observed in *Wrn*^*−/−*^ MEFs even though these cells display decreased levels of NADPH and GSH and increased mitochondrial respiration, when compared to *WT* MEFs (Fig. S10, Supporting Information), possibly suggesting that MEFs can better adapt to the metabolic shift induced by the loss of WRN.

### Werner syndrome protein is required for proper expression of the transcription factor hypoxia-inducible factor 1 (HIF1)α

**Figure 5:**
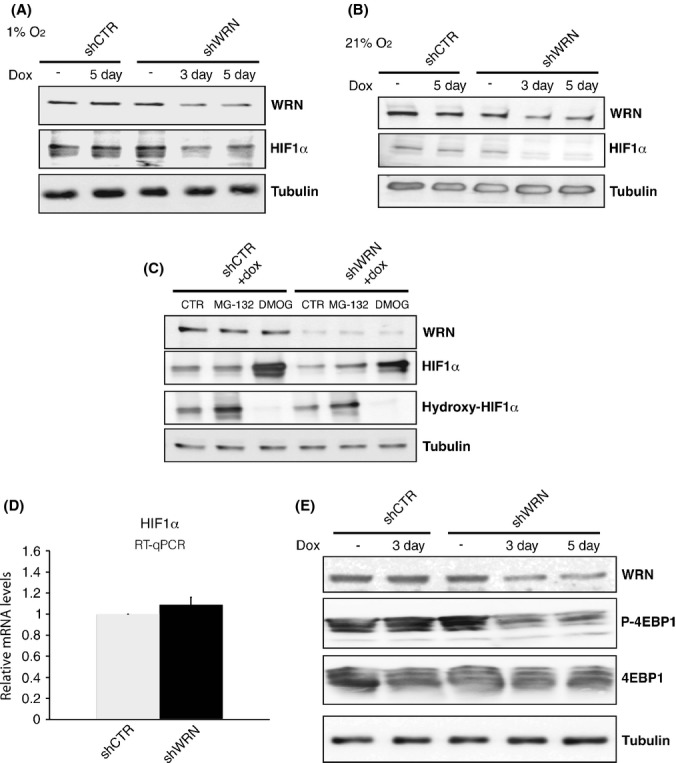
Werner syndrome protein (WRN) knockdown affects the levels of hypoxia-inducible factor 1 (HIF1α). Nuclear extracts were prepared from Hela cells transduced with lentiviral vectors for the expression of shRNAs against WRN or green fluorescence protein (GFP) (CTR) that were grown in the absence or presence of doxycycline (+dox) for 3 or 5 days under atmosphere of 1% (A) or 21% (B) oxygen, and analyzed by immunoblotting using antibodies against WRN, HIF1α and α-tubulin as loading control. (C) Nuclear extracts from shCTR and shWRN Hela cells treated with or without MG-132 or dimethyloxalylglycine as indicated were analyzed by immunoblotting with antibodies specific to hydroxylated HIF1 α (hydroxyl-HIF1α) or total HIF1α (D), Reverse transcriptase–quantitative polymerase chain reaction (RT–qPCR) analysis of mRNA steady-state levels for HIF1α in Hela cells grown in 1% oxygen 3 days after WRN knockdown. mRNA expression levels were normalized to tubulin mRNA and are shown as relative to shCTR cells. RNA was isolated from at least three biological samples of Hela cells with shCTR or shWRN, and RT–qPCR assays were carried out in triplicate samples. The graph and statistics were generated using Excel. (E) Extracts were prepared from Hela cells transduced with lentiviral vectors for the expression of shRNAs against WRN or GFP (CTR) that were grown in the absence or presence of doxycycline (+dox) for the indicated days, and analyzed by immunoblotting using antibodies against WRN, phosphorylated 4E binding protein 1 (4EBP1) (P-4EBP1), total 4EBP1, and α-tubulin as loading control.

Cancer cells, particularly under hypoxic conditions, mount an adaptive response to ROS accumulation that is generally implemented by cellular programs including the stabilization of the transcription factor HIF1 (Semenza, 2011). Hypoxia-inducible factor 1 modulates the expression of genes that regulate glycolysis and repress mitochondrial respiration in a coordinate manner, thereby attenuating ROS production (Kirito *et al*., 2009). To determine whether ROS accumulation after WRN knockdown was associated with compromised HIF1, we examined the levels of HIF1α, the regulated subunit of the HIF1 complex, in nuclear extracts of HeLa cells expressing shWRN grown under 1% O_2_. We observed reduced levels of HIF1α in Hela cells expressing inducible shRNAs after WRN knockdown compared to control cells, indicating that a major regulator of the adaptive response to oxidative stress is influenced by WRN (Figs 5A and S11, Supporting Information). Interestingly, shWRN cells grown in 21% O_2_ also showed reduced HIF1α levels compared to control cells (Fig. 5B), indicating that WRN depletion affects both basal and activated levels of HIF1α. The observation that the levels of HIF1α increased in WRN-depleted cells shifted from 21% to 1% O_2_ (Fig. S11, Supporting Information), further suggests that WRN depletion does not prevent the stabilization of HIF1α induced by hypoxia.

We sought to determine the molecular mechanism by which depletion of WRN influences the levels of HIF1α. The regulation of HIF1α is complex, and several mechanisms including transcription, translation, post-translational modification, protein–protein interactions, and degradation have been shown to affect the levels of this transcription factor (Yee Koh *et al*., 2008). Because destabilization through proline hydroxylation and subsequent degradation through the proteasome is a major mechanism regulating HIF1α levels, we first examined whether WRN depletion affects HIF1α protein stability. We measured the extent of HIF1α hydroxylation in shWRN and control cells treated with the proteasome inhibitor MG-132 to prevent hydroxylated HIF1α to be degraded or with dimethyloxalylglycine (DMOG) to inhibit prolyl hydroxylase activity. WRN-depleted cells treated with MG132 did not display an increase in the levels of hydroxylated HIF1α compared to control (Fig. 5C). Moreover, DMOG-treated shWRN cells show lower overall levels of HIF1α than control cells. Collectively, these results suggest that WRN depletion does not affect the stability of HIF1α. To determine whether WRN binds to HIF1α we performed reciprocal immunoprecipitation assays and probed for interactions between these two proteins by immunoblotting. These experiments did not reveal a detectable interaction between WRN and HIF1α (not shown). Next, to assess whether WRN regulates the transcription or mRNA stability of HIF1α, we measured steady-state mRNA levels by RT-qPCR and did not observe any reduction in HIF1α mRNA in shWRN cells as compared to controls (Fig. 5D). We then tested whether WRN depletion might affect the translation of HIF1α. Hypoxia-inducible factor 1α translation is controlled through a mechanism that involves hyperphosphorylation of the eukaryotic initiation factor 4E binding protein 1 (4EBP1) (Laughner *et al*., 2001; Thomas *et al*., 2006; Duvel *et al*., 2010). Hypophosphorylated 4EBP1 binds to and inactivates the eukaryotic initiation factor 4E (eIF4E), thereby inducing translational inhibition (Pause *et al*., 1994). We assessed the phosphorylation status of 4EBP1 in WRN-depleted and control cells by western blotting. These experiments demonstrated that 4EBP1 is rapidly dephosphorylated after WRN knockdown (Fig. 5E), suggesting that HIF1α levels are reduced in WRN-depleted cells through a mechanism that involves 4EBP1-mediated translational repression.

### Treatment with antioxidant improves growth and reduces senescence of WRN-knockdown cancer cells

**Figure 6:**
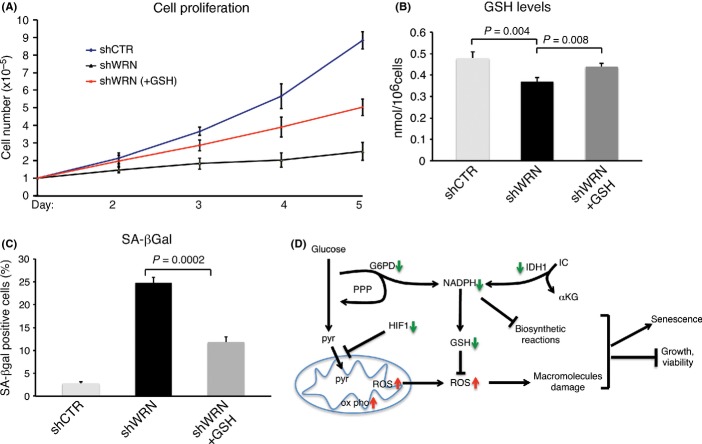
Supplementation with reduced glutathione (GSH) ameliorates growth and reduces senescence of Werner syndrome protein (WRN)-depleted cells. (A) Hela cells transduced with lentiviral vectors for the expression of shRNAs against WRN were grown in normal media or media supplemented with 2 mM GSH. Cells were seeded at a low density in an atmosphere of 1% oxygen, and the medium was changed every 2 days. Values represent the mean ± the standard deviation of three experiments (*n* = 3). (B) GSH levels was measured in Hela cells transduced with lentiviral vectors for the expression of shRNAs against WRN or shGFP that were grown in the presence of doxycycline (+dox) for 3 days in an atmosphere of 1% oxygen. When indicated, GSH was added to the culture media. (C) Detection of senescence-associated β-galactosidase (SA-β-gal) activity. Hela cells transduced with lentiviruses for the conditional expression of shRNAs targeting WRN or green fluorescence protein (GFP) were grown for 5 days after the addition of DOX and stained for SA-β-gal activity as previously described (Li *et al*., 2008). Values are the mean ± the standard deviation of three independent experiments (*n* = 3) carried out in duplicates, in which 500 cells were scored for SA-β-galactosidase. Student’s *t*-test was used to evaluate the differences in means between the two groups. (D) Hypothetical model: downregulation or loss of WRN compromises the cellular antioxidant system by reducing the levels of NADPH-producing enzymes glucose 6 phosphate dehydrogenase and isocitrate dehydrogenase. Reduced levels of NADPH, a source of reducing power, will affect cellular anabolism. In cancer cells, concomitant to altered mitochondrial function, this metabolic shift leads to the accumulation of reactive oxygen species, which causes damage to macromolecules and impairs cell viability.

Our data show that WRN knockdown in Hela cells results in the production of ROS and oxidative DNA damage. To determine whether a reduced capacity to detoxify ROS contributed to limiting the proliferation and increasing the senescence of these cells, we tested whether supplementation with antioxidant rescues these phenotypes. Treatment of Hela cells grown under the atmosphere of 1% O_2_ with GSH prior to WRN depletion improved proliferation and was accompanied by a remarkable reduction in senescence (Fig. 6A–C), indicating that oxidative stress contributes to the onset of both phenotypes. However, supplementation with glutathione did not mitigate DNA damage (Fig. S12, Supporting Information), suggesting that antioxidant might rescue other cellular processes affected by ROS accumulation.

## Discussion

Many cancer cells express relatively high levels of WRN, and its depletion results in growth suppression [this study, (Opresko *et al*., 2007)]. These findings suggest that WRN is important for maintaining the proliferative state of cancer cell. In this study, we provide evidence that WRN supports cancer cell proliferation by regulating the pathways critical for powering macromolecular synthesis and limiting oxidative stress. The metabolic requirements of cancer cells are dramatically altered compared to normal cells. Cancer cells adopt a striking metabolic shift, known as ‘Warburg effect’, which limits mitochondrial respiration and redirects glucose catabolism into the pentose phosphate pathway. This shift is necessary to support the increased requirements of proliferation of these cells through the generation of metabolic intermediates and reducing equivalents necessary for *de novo* synthesis of nucleotides and for regenerating the cellular levels of GSH required to control ROS (Cairns *et al*., 2011). Downregulation of WRN results in a decrease in the levels of metabolic enzymes required for the production of two powerful antioxidants, NADPH and GSH (Fig. 3), and is accompanied by significant alterations in mitochondrial output and ROS accumulation (Fig. 4). It has been suggested that low levels of ROS are beneficial for cancer cells function, as they induce an adaptive response that enables these cells to grow and proliferate (Sena & Chandel, 2012). However, increased basal levels of ROS render cancer cells more vulnerable to conditions that further increase oxidant production or weaken the antioxidant defense system of the cell. Thus, the formation of a debilitated antioxidant system accompanied by an increased mitochondrial respiration that results from WRN knockdown poses a significant stress in cancer cells and renders them susceptible to increased oxidative damage to DNA and other macromolecules. This outcome is likely to contribute to the reduced cell proliferation and the increase in cells stained for SA-β-gal after WRN depletion (Fig. S4). The cellular pathways affected by WRN depletion are also expected to negatively influence biosynthetic reactions requiring reducing equivalents in the form of NADPH, resulting in limited supply of macromolecules necessary for vigorous cell growth and proliferation.

Reactive oxygen species are thought to play a physiological role in normal cells by regulating signaling pathways (Finkel, 2012). However, maintaining a balanced intracellular redox homeostasis is vital for any cell, as increases in ROS production can contribute to oxidative damage to a variety of macromolecules, thus impairing cell function. Preventing oxidative stress is heavily dependent on antioxidant molecules, which allow the cell to manage fluctuations in ROS levels. Because WRN knockdown alters the levels of proteins involved in the antioxidant system, we examined how normal cells cope with the loss of WRN. The analysis of *Wrn*^*−/−*^ MEFs demonstrates that these cells have reduced NADPH and GSH levels and higher levels of respiration, as compared to matched *WT* MEF controls (Fig. S10A–C). However, superoxide levels were not increased and we did not observe a detectable increase in oxidative DNA damage in low-passage *Wrn*^*−/−*^ MEFs (Fig. S10D,E), suggesting that these cells can overcome the metabolic alterations induced by the loss of WRN. The differential resistance to oxidative stress between normal and cancer cells in response to acute WRN knockdown suggests that drugs targeting WRN could be used to hypersensitize tumor cells. Nevertheless, we cannot rule out that, even though normal cells have a better adaptive response and are more tolerant than cancer cells to oxidative stress, loss of WRN function may foster an oxidative environment that results in a slow but steady accumulation of oxidative damage to macromolecules. This might not dramatically affect cell homeostasis on the short term, but it could contribute to premature senescence over a long period of time, which is consistent with a disease like WS characterized by the progressive onset of aging pathologies after puberty.

The transcription factor HIF1 is an important regulator of metabolic processes that influence glucose utilization and mitochondrial function, especially in cancer cells. Thus, a decrease in HIF1 levels could be responsible for the metabolic shift induced by the depletion of WRN. However, attempts to reconstitute metabolic output in WRN-depleted cells by ectopic expression of HIF1α were unsuccessful (data not shown), suggesting that other regulatory pathways affected by WRN are critically involved in eliciting the metabolic shift. Our data show that WRN depletion results in the hypophosphorylation of the translational inhibitor 4EBP1, a factor that is crucial for HIF1α mRNA translation. Recent studies have demonstrated that 4EBP1 preferentially controls the translation of a class of mRNAs with a 5 prime terminal polypyrimidine (5′TOP) or TOP-like sequence (Thoreen *et al*., 2012), suggesting that selective translational inhibition by 4EBP1 may play an important role in the cellular changes induced by WRN depletion. Preliminary analyses indicate that the decrease in the abundance of other metabolic proteins including G6PD and IDH1 after WRN depletion does not arise from changes in mRNA steady-state levels (data not shown), possibly implicating a mechanism of translational inhibition. Whether the mammalian target of rapamycin (mTOR) kinase, a major regulator of 4EBP1 phosphorylation (Yee Koh *et al*., 2008), plays any role in this process remains to be determined.

Werner syndrome protein has been linked to a variety of nuclear transactions including transcription, replication, repair, and recombination (Rossi *et al*., 2010), but its precise role in any of these processes remains unclear. Several studies demonstrated functional interactions between WRN and components of the telomere protection complex termed shelterin (Opresko *et al*., 2002, 2005; Machwe *et al*., 2004; Li *et al*., 2009) and have shown that WRN contributes to the maintenance of telomere length homeostasis in human cells (Crabbe *et al*., 2004; Opresko *et al*., 2004; Li *et al*., 2008). Telomeric sequences, which are rich in guanine residues, are particularly sensitive to oxidative stress, and mitochondria dysfunction has been functionally linked to DNA damage at telomeres and telomere shortening (Passos *et al*., 2007; Hewitt *et al*., 2012). The alterations in telomere homeostasis associated with WRN deficiency include the stochastic shortening of the G-rich telomeric strand (Crabbe *et al*., 2004). This phenotype has been attributed to defects in the synthesis of the lagging strand of the telomere, although accumulation of oxidative guanine lesions followed by strand breaks can contribute to telomere shortening preferentially at the lagging strand (Wang *et al*., 2010). Thus, our data suggest a potential link between oxidative stress and altered telomere length homeostasis in the context of WRN deficiency.

In conclusion, our study provides evidence for a novel link between WRN depletion and metabolic alterations leading to oxidative stress. Although the precise mechanism underlying these processes remains to be determined, it is likely that altered metabolism plays an important role in the onset of the phenotypes caused by the loss of WRN function.

## Experimental procedures

### Cell lines

Hela, MCF-7, and AG14F/20 fibroblasts were purchased from ATCC. *Wrn*^*−/−*^ and *Wrn*^+/+^ MEFs were a kind gift from Brad Johnson (University of Pennsylvania). The cells were cultured in Dulbecco modified essential medium (DMEM, containing l-glutamine and 4.5 g L^−1^ glucose, without sodium pyruvate) supplemented with 10% fetal calf serum (FBS) and 1% penicillin/streptomycin and maintained at 37 °C in a humidified incubator at 5% CO_2_ under atmospheric (21%) or hypoxic (1%) oxygen.

### Inducible lentiviral shRNA vectors

To generate lentiviral vectors for the conditional expression of shRNAs targeting WRN or GFP, complementary oligonucleotides were annealed and cloned into a pENTT-miRc2 vector. Target sequences are as follows: WRN-a: 5′ TGAAGAGCAAGTTACTTGCCT-3′; WRN-b:AGGTCCAACAATCATCTACTGT-3′; GFP-a: 5′-CGCAAGTCGACCCTGAGTTCA-3′; GFP-b:5′-GTTCATCTGCACCACCGGCTT-3′. The shRNA sequences for WRN and GFP control are specific for their respective targets and have no significant homology to any known human gene. miR-shRNA vectors were then generated by *in vitro* recombination between pENTT-miRc2 and pSLIK-Neo or pSLIK-Hyg using the Gateway LR Clonase Enzyme Mix Kit (Invitrogen, Carlsbad, CA, USA). Recombinant lentiviruses were produced as previously described (44). For lentiviral infections, the viral supernatant was added to cultured cells for 6 h, then the supernatant was removed, and the cells were washed twice with phosphate-buffered saline (PBS). Cells were incubated in DMEM containing 10% FBS at 37 °C for 1 day and then subjected to selection in DMEM supplemented with 400 μg mL^−1^ geneticin (G418) and 200 μg mL^−1^ hygromycin for 7 days. One microgram per millilitre doxycycline (dox) was added to the media to induce shRNAs expression. The level of downregulation of the target proteins was estimated by western blotting using α-tubulin as a loading control.

### Immunoblotting

Extracts were prepared and immunoblot analyses were performed using antibodies against WRN (BL1309; Bethyl Inc., Montgomery, TX, USA), HIF1α (610958; BD Biosciences, San Jose, CA, USA), IDH1 (GTX14484; GeneTex, Irvine, CA, USA), G6PD (GTX101212; GeneTex), TKTL1 (GTX109459; GeneTex), and α-tubulin (SC-5286; Santa Cruz Biotechnology Inc., Dallas, TX, USA), p53 (SC-126; Santa Cruz Biotechnology Inc.), p21 (C-19; Santa Cruz Biotechnology Inc.), p16 (C-20; Santa Cruz Biotechnology Inc.), 4E-BP1 clone 53H11 (rabbit mAb #9644; Cell Signaling), 4E-BP1 (#9452; Cell Signaling, Beverly, MA, USA), phospho-4E-BP1 Thr37/46 clone 236B4 (rabbit mAb #2855; Cell Signaling). Anti-rabbit and anti-mouse immunoglobulin G horseradish peroxidase-coupled antibodies were purchased from Promega. To assess HIF1α protein stability, shCTR and shWRN Hela cells were cultured in medium with 1.0 μg mL^−1^ dox for 5 days in 1% O_2_ and then treated with 10 μm carbobenzoxy-l-leucyl-l-leucyl-l-leucinal (MG-132) for 1 h or 1 mm DMOG for 4 h. Nuclear extracts were prepared and the levels of hydroxylated-HIF1α and total HIF1α were determined using antibodies against hydroxylated HIF1α (rabbit mAb #3434P; Cell Signaling) and HIF1α, respectively.

#### Biochemical methods

Extract preparation and immunoblotting were carried out as previously described (Li *et al*., 2008, 2011). For the 2D DIGE experiments, frozen cell pellets were sent for analysis to Applied Biomics Inc. (Hayward, CA, USA). Mitochondria oxygen consumption was measured using a Seahorse XF24 analyzer (Seahorse Bioscience, North Billerica, MA, USA), and more details are provided in Supporting Information. Measurements of superoxide radical anions, peroxides, NADPH, GSH, and mitochondria membrane potential were carried out using standard assay conditions, and full methods are provided in Data S1 (Supporting Information).

#### Analysis of mitochondria morphology and function

For transmission electron microscopy, cells were fixed, processed, and analyzed on a JSM electron microscope (JEOL USA, Inc., Pleasanton, CA, USA) at the USC Keck School of Medicine Electron Microscopy Core Facility. For the determination of oxygen consumption rates (OCR), cells were cultured on Seahorse XF-24 plates at a density of 5 × 10^4^ cells per well and analyzed as described in details in Data S1.

### Analysis of mitochondrial DNA copy number

Mitochondrial DNA (mtDNA) copy number was quantified by qPCR from total DNA isolated using the QIAamp DNA mini kit (Cat#51304, Valencia, CA, USA) using primer pairs for the genomic β2-microglobulin gene (5′-TGCTGTCTCCATGTTTGATGTATCT-3′; 5′-TCTCTGCTCCCCACCTCTAAGT-3′) and mitochondrial tRNA^Leu(UUR)^ gene (5′-CACCCAAGAACAGGGTTTGT-3′: 5′-TGGCCATGGGTATGTTGTTA-3′). All the samples were measured in triplicates, and qPCR results were confirmed by four independent experiments. Data obtained by qPCR were analyzed by the Pfaffl method.

Additional experimental details are described in Data S1.

## Supporting Information

**Fig. S1** Western blot analysis showing levels of WRN in normal diploid human fibroblasts (NDF) and NDF transduced with lentiviral vector for the conditional expression of shRNA targeting GFP (shCTR) and WRN (shWRN) at different days (d) after addition of doxycycline (1 μg mL^−1^). **Fig. S2** (A) 2D-DIGE gel images showing spots (circles) that were picked for MS analysis. (B) Proteins identified by 2D-DIGE analysis, showing relative levels at 3 and 5 days after WRN knockdown compared to control (shCTR). **Fig. S3** Extracts prepared from *Wrn*^*−/−*^ and *Wrn*^*+/+*^ MEFs (passage 4) were analyzed by immunoblotting using antibodies against the indicated proteins. **Fig. S4** (A) Growth curves of Hela and MCF7 cells expressing shRNAs for WRN or GFP. One microgram per millilitre doxycycline was added to the media and the growth rate of each cell line was measured by counting viable cells every 2 days. Cells were seeded at a low density, and the medium was changed every 2 days. Values represent the mean ± the standard deviation of three experiments (*n* = 3). (B) Detection of SA-β-gal activity. Hela and MCF7 cells transduced with lentiviruses for the conditional expression of shRNAs targeting WRN or GFP were grown for 3, 6, 9, or 12 days after addition of DOX and were stained for SA-βgal activity as previously described {Li, 2011 #778}. Values are the mean ± the standard deviation of three independent experiments (*n* = 3) carried out in duplicates in which 500 cells were scored for SA-β galactosidase. Student’s *t* test was used to evaluate differences in means between two groups, and *P* < 0.05 was considered statistically significant. (C) Cell cycle profile of Hela cells transduced with lentiviral vectors for the conditional expression of shRNA targeting WRN (shWRN) or GFP (shCTR) before and at days 1, 2 and 3 after induction with doxycycline. **Fig. S5** WRN knockdown in MCF7 cells alters the levels of metabolic enzymes. **Fig. S6** Representative Western blots loaded with serially diluted samples used to assess the levels of G6PD, IDH1, TKTL1, and HIF1α in shCTR and shWRN cells, as shown in the tables of Figures 2, 5, S5 and S8. **Fig. S7** (A) Western blots showing levels of G6PD, IDH1 and TKTL1 in HeLa cells grown in 1% serum before and at 3 and 5 days after induction of shRNAs against WRN or GFP (shCTR). (B) siRNA-mediated WRN knockdown in Hela cells recapitulates the changes in metabolic enzymes observed after expression of shRNAs targeting WRN. **Fig. S8** Changes in the levels of metabolic enzymes in WRN knockdown cancer cells grown under hypoxia. **Fig. S9** (A) GSH levels were measured in Hela cells transduced with lentiviral vectors for the expression of shRNAs against GFP or WRN that were grown in 1% serum in the absence or presence of doxycycline (+dox) for 3 days. Each data point represents the mean ± SD of three biological replicates, and *P* values were calculated by two-tailed Student’s *t* test. (B) Representative experiment showing oxygen consumption rates in WRN knockdown and control (shCTR) Hela cells. OCR was determined using Seahorse XF-24 Metabolic Flux Analyzer. Vertical lines indicate time of addition of mitochondrial inhibitors: oligomycin (4 μm; ATP synthase inhibitor), FCCP (1 μm; uncoupler), or rotenone (1 μm; complex I inhibitor). In the experiment shown, samples of Hela cells transduced with vector for the expression of shWRN before and after induction with doxycycline as well as control cells transduced with vector for the expression of shGFP (shCTR) after induction with doxycycline. WRN knockdown cells after shRNA induction (solid black line) display higher state III and uncoupled (after the addition of FCCP) rates of mitochondrial respiration than uninduced Hela with shWRN (grey dashed line) and doxycycline induced control cells (shCTR) (grey solid line). (C) Representative confocal microscopy images of Hela cells transduced with lentiviruses for the conditional expression of shRNAs targeting WRN or GFP (shCTR) detecting oxidized nucleoside-8-hydroxy-2′-deoxyguanosine (8HO-dG) or phosphorylated H2AX (γH2AX) in the indicated sample. **Fig. S10** Altered metabolism in *Wrn* knockout MEFs. **Fig. S11** Reduced levels of HIF1α after WRN knockdown in cancer cells. **Fig. S12** Hela cells transduced with lentiviral vectors for the expression of shRNAs against WRN or GFP (shCTR) were grown in the absence or presence of doxycycline (+dox) and in normal media or media supplemented with 2 mm GSH. **Table S1** Gene ontology enrichment analysis software was utilized to assign proteins to biological processes.

**Data S1** Experimental procedures.
