# Supplementary material for: Downregulation of the Werner syndrome protein induces a metabolic shift that compromises redox homeostasis and limits proliferation of cancer cells
Source: Aging Cell. 2013 Dec 1;13(2):367–78. doi: 10.1111/acel.12181 (PMC3999508; doi:10.1111/acel.12181)
Supplement: Supplementary file 1 — Fig. S1 Western blot analysis showing levels of WRN in normal diploid human fibroblasts (NDF) and NDF transduced with lentiviral vector for the conditional expression of shRNA targeting GFP (shCTR) and WRN (shWRN) at different days (d) after addition of doxycycline (1 μg mL−1). Fig. S2 (A) 2D-DIGE gel images showing spots (circles) that were picked for MS analysis. (B) Proteins identified by 2D-DIGE analysis, showing relative levels at 3 and 5 days after WRN knockdown compared to control (shCTR). Fig. S3 Extracts prepared from Wrn−/− and Wrn+/+ MEFs (passage 4) were analyzed by immunoblotting using antibodies against the indicated proteins. Fig. S4 (A) Growth curves of Hela and MCF7 cells expressing shRNAs for WRN or GFP. One microgram per millilitre doxycycline was added to the media and the growth rate of each cell line was measured by counting viable cells every 2 days. Cells were seeded at a low density, and the medium was changed every 2 days. Values represent the mean ± the standard deviation of three experiments (n = 3). (B) Detection of SA-β-gal activity. Hela and MCF7 cells transduced with lentiviruses for the conditional expression of shRNAs targeting WRN or GFP were grown for 3, 6, 9, or 12 days after addition of DOX and were stained for SA-βgal activity as previously described {Li, 2011 #778}. Values are the mean ± the standard deviation of three independent experiments (n = 3) carried out in duplicates in which 500 cells were scored for SA-β galactosidase. Student’s t test was used to evaluate differences in means between two groups, and P < 0.05 was considered statistically significant. (C) Cell cycle profile of Hela cells transduced with lentiviral vectors for the conditional expression of shRNA targeting WRN (shWRN) or GFP (shCTR) before and at days 1, 2 and 3 after induction with doxycycline. Fig. S5 WRN knockdown in MCF7 cells alters the levels of metabolic enzymes. Fig. S6 Representative Western blots loaded with serially diluted samples used to a [file acel0013-0367-sd1.pdf]

Supplementary Information for "*Downregulation of the Werner syndrome protein induces a metabolic shift that compromises redox homeostasis and limits proliferation in cancer cells"*

Supplementary Material

Supplementary Figure S1. Western blot analysis showing levels of WRN in normal diploid human fibroblasts (NDF) and NDF transduced with lentiviral vector for the conditional expression of shRNA targeting GFP (shCTR) and WRN (shWRN) at different days (d) after addition of doxycycline (1μg/ml). α-tubulin was used as loading control.

Supplementary Figure S2. (**A**), 2D-DIGE gel images showing spots (circles) that were picked for MS analysis. (**B**), Proteins identified by 2D-DIGE analysis, showing relative levels at 3 and 5 days after WRN knockdown compared to control (shCTR). Quantitative analysis of protein spots between different samples was carried out using DeCyder Differential In-gel Analysis (DIA) software.

Supplementary Figure S3. Extracts prepared from $Wrn^{-/-}$ and $Wrn^{+/+}$ MEFs (passage 4) were analyzed by immunoblotting using antibodies against the indicated proteins. For this analysis, MEFs were grown under 1% O_2 grown since under atmospheric oxygen we observed extensive loss of $Wrn^{-/-}$ MEFs viability. α–tubulin was used as a loading control. Quantification of chemiluminescent signals from western blots of three biological replicates was carried out using the Image Analyzer LAS-4000 (*Fujifilm* Life-Science) *and mean relative value* ($Wrn^{+/+}$ *set at 100 A.U.)* ± SD are shown. α-tubulin was used as normalizing factor.

Supplementary Figure S4. (**A**), Growth curves of Hela and MCF7 cells expressing shRNAs for WRN or GFP. 1.0μg/ml doxycycline was added to the media and the growth rate of each cell line was measured by counting viable cells every 2 days. Cells were seeded at a low density, and the medium was changed every 2 days. Values represent the mean ± the standard deviation of three experiments (*n*=3). (**B**), Detection of SA-β-gal activity. Hela and MCF7 cells transduced with lentiviruses for the conditional expression of shRNAs targeting WRN or GFP were grown for 3, 6, 9, or 12 days after addition of DOX and were stained for SA-βgal activity as previously described {Li, 2011 #778}. Values are the mean ± the standard deviation of three independent experiments (*n*=3) carried out in duplicates in which 500 cells were scored for SA-β galactosidase. Student's *t* test was used to evaluate differences in means between two groups, and $P<0.05$ was considered statistically significant. (**C**), Cell cycle profile of Hela cells transduced with lentiviral vectors for the conditional expression of shRNA targeting WRN (shWRN) or GFP (shCTR) before and at days 1, 2 and 3 after induction with doxycycline. FACS analysis of Hela cells stained with propidium iodine was carried out using three biological replicates and data were analyzed using the Watson Pragmatic model.

Supplementary Figure S5. WRN knockdown in MCF7 cells alters the levels of metabolic enzymes. (**A**), Western blot analysis showing the abundance of WRN, p21 and p16 in control and shWRN cells before and after induction with doxycycline. α-tubulin was used as normalizing factor. (**B**), Representative Western blots showing levels of G6PD, IDH1 and TKTL1 in MCF7 cells before and at 3 and 5 days after induction of shRNAs against WRN or GFP (shCTR). Quantification of chemiluminescent signals from western blots of three biological replicates was carried out using the Image Analyzer LAS-4000 (*Fujifilm* Life-Science) as described in the supplementary experimental procedures section and mean relative value [shCTR-dox set at 100

arbitrary units (a.u.)] ± SD are shown. α-tubulin was used as normalizing factor. *** denotes a P value <0.00001.

Supplementary Figure S6. Representative Western blots loaded with serially diluted samples used to assess the levels of G6PD, IDH1, TKTL1, and HIF1α in shCTR and shWRN cells, as shown in the tables of Figures 2, 5, S5 and S8.

Supplementary Figure S7. (**A**), Western blots showing levels of G6PD, IDH1 and TKTL1 in HeLa cells grown in 1% serum before and at 3 and 5 days after induction of shRNAs against WRN or GFP (shCTR). (**B**), siRNA-mediated WRN knockdown in Hela cells recapitulates the changes in metabolic enzymes observed after expression of shRNAs targeting WRN. Hela cells were transfected with siRNAs targeting WRN or scrambled siRNAs and analyzed four days after transfection by immunoblotting using antibodies against the indicated proteins. α-tubulin was used as a loading control. Quantification of chemiluminescent signals from western blots was carried out using the Image Analyzer LAS-4000 (*Fujifilm* Life-Science) and relative values (siCTR set at 100 arbitrary units (a.u.)) are shown. α-tubulin was used as normalizing factor.

Supplementary Figure S8. Changes in the levels of metabolic enzymes in WRN knockdown cancer cells grown under hypoxia. (**A**), Western blot analysis showing levels of G6PD, IDH1, and TKTL1 in Hela cells grown under 1% O_2 before and at 3 and 5 days after induction of shRNAs against WRN (shWRN) or GFP (shCTR). α–tubulin was used as a loading control. (**B**), Western blot analysis of showing levels of G6PD, IDH1 and TKTL1 in MCF7 cells grown under 1% O_2 before and at 3 and 5 days after induction of shRNAs against WRN (shWRN) or GFP (shCTR). α–tubulin was used as a loading control. (**C**), Quantification of chemiluminescent signals from western blots was carried out using the Image Analyzer LAS-4000 (*Fujifilm* Life-

Science) and relative values (shCTR-dox set at 100 a.u.) are shown. α-tubulin was used as normalizing factor.

Supplementary Figure S9. (**A**), GSH levels were measured in Hela cells transduced with lentiviral vectors for the expression of shRNAs against GFP or WRN that were grown in 1% serum in the absence or presence of doxycycline (+dox) for 3 days. Each data point represents the mean ± SD of three biological replicates, and P values were calculated by two-tailed Student's t test. (**B**), Representative experiment showing oxygen consumption rates in WRN knockdown and control (shCTR) Hela cells. OCR was determined using Seahorse XF-24 Metabolic Flux Analyzer. Vertical lines indicate time of addition of mitochondrial inhibitors: oligomycin (4 μM; ATP synthase inhibitor), FCCP (1 μM; uncoupler), or rotenone (1 μM; complex I inhibitor). In the experiment shown, samples of Hela cells transduced with vector for the expression of shWRN before and after induction with doxycycline as well as control cells transduced with vector for the expression of shGFP (shCTR) after induction with doxycycline. WRN knockdown cells after shRNA induction (solid black line) display higher state III and uncoupled (after the addition of FCCP) rates of mitochondrial respiration than uninduced Hela with shWRN (grey dashed line) and doxycycline induced control cells (shCTR) (grey solid line). (**C**), Representative confocal microscopy images of Hela cells transduced with lentiviruses for the conditional expression of shRNAs targeting WRN or GFP (shCTR) detecting oxidized nucleoside-8-hydroxy-2'-deoxyguanosine (8HO-dG) or phosphorylated H2AX (γH2AX) in the indicated samples. Quantitative analyses of these data are shown in Figure 4.

Supplementary Figure S10. Altered metabolism in *Wrn* knockout MEFs. (**A**), NADPH and (**B**), GSH levels were measured in $Wrn^{+/+}$ and $Wrn^{-/-}$ MEFs grown under 1% oxygen as described in Materials. Each data point represents the mean ± SD of three independent experiments, and P

values were calculated by two-tailed Student's t test. (**C**), Representative data of respiration analysis in *Wrn*$^{-/-}$ and WT MEFs. OCR was determined using Seahorse XF-24 Metabolic Flux Analyzer. Vertical lines indicate time of addition of mitochondrial inhibitors: oligomycin (4 μM), FCCP (1 μM), or rotenone (1 μM). *Wrn*$^{-/-}$ MEFs display higher basal and uncoupled (after the addition of FCCP) rates of mitochondrial respiration than WT MEFs. (**D**), Superoxide anions radical generated by the mitochondria in *Wrn*$^{-/-}$ and *Wrn*$^{+/+}$ MEFs were measured using MitoSOX Red and analyzed by flow cytometry. Histogram from representative experiment is shown.

Supplementary Figure S11. Reduced levels of HIF1α after WRN knockdown in cancer cells. (**A**), Quantification of chemiluminescent signals from western blots (as shown in Figure 5A) of three biological replicates was carried out using the Image Analyzer LAS-4000 (*Fujifilm* Life-Science) and mean relative values (shCTR-dox set at 100 a.u.) ± SD are shown. α-tubulin was used as normalizing factor. *** denotes a P value <0.00001. (**B**) Hela cells grown under 1% or 21% oxygen were transfected with siRNAs targeting WRN or scrambled siRNAs (SMARTpool, Dharmacon) and analyzed four days after transfection by immunoblotting using antibodies against WRN, HIF1α and α-tubulin (loading control). (**C**), Nuclear extracts were prepared from MCF7 cells transduced with lentiviral vectors for the expression of shRNAs against WRN or GFP (shCTR) that were grown in 1% (top panel) or 21% (bottom panel) oxygen in the absence or presence of doxycycline (+dox) for 3 and 5 days, and analyzed by immunoblotting using antibodies against WRN, HIF1α and α-tubulin (loading control). (**D**), Nuclear extracts were prepared from Hela cells transduced with lentiviral vectors for the expression of shRNAs against WRN or GFP (CTR) that were grown in 1% serum in the absence or presence of doxycycline (+dox) for 3 or 5 days, and analyzed by immunoblotting using antibodies against WRN, HIF1α and α-tubulin as loading control.

Supplementary Figure S12. Hela cells transduced with lentiviral vectors for the expression of shRNAs against WRN or GFP (shCTR) were grown in the absence or presence of doxycycline (+dox) and in normal media or media supplemented with 2 mM GSH. The oxidized nucleoside 8 hdryoxy-2'-deoxyguanosine (8HO-dG) and phosphorylated histone H2AX (γH2AX) were detected by immunofluorescence microscopy. Quantification of nuclear fluorescence per cell for each sample was carried out using Image J software and data were plotted using GraphPad software.

Table S1. Gene Ontology enrichment analysis software was utilized to assign proteins to biological processes. A brief explanation of the parameter included in each column is provided at the bottom of the table.

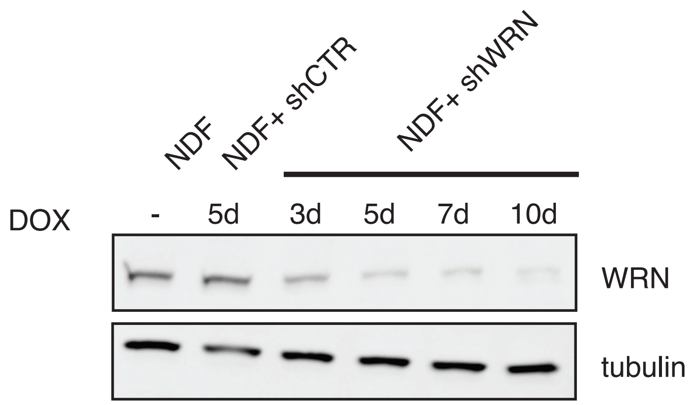

Figure S1
Li et al.

(A)

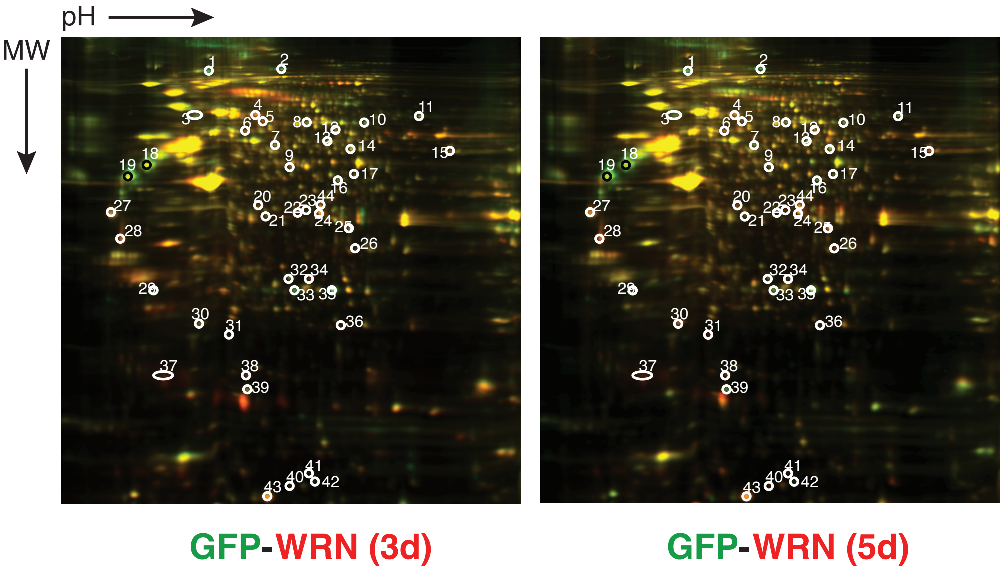

(B)

SPOT #	Protein ID	FOLD CHANGES shWRN /shGFP 3days	shWRN/shGFP 5 days
1	collagen alpha-1(VI) chain precursor [Homo sapiens]	-4.13	-4.53
2	collagen alpha-2(VI) chain isoform 2C2 precursor [Homo sapiens]	-3.86	-4.43
3	lamin-B1 isoform 1 [Homo sapiens]	-1.44	-1.64
4	serum albumin [Bos indicus]	1.87	1.51
5	eukaryotic translation initiation factor 3 subunit D [Homo sapiens]	2.42	1.56
6	copine-3 [Homo sapiens]	1.39	1.56
7	tryptophanyl-tRNA synthetase, cytoplasmic isoform a [Homo sapiens]	-1.40	-1.92
8	prelamin-A/C isoform 2 [Homo sapiens]	-1.52	-1.75
9	Eukaryotic translation elongation factor 1 gamma [Homo sapiens]	1.52	1.03
10	heterogeneous nuclear ribonucleoprotein L, isoform CRA_a [Homo sapiens]	-1.63	-1.57
11	transketolase variant [Homo sapiens]	-1.56	-1.77
12	Hpast [Homo sapiens]	1.22	1.57
13	glucose-6-phosphate 1-dehydrogenase isoform b [Homo sapiens]	-1.24	-1.89
14	FSCN1 protein [Homo sapiens]	-1.26	-1.64
15	ATP synthase subunit alpha, mitochondrial precursor [Homo sapiens]	1.09	1.84
16	Isocitrate dehydrogenase 1 (NADP+), soluble [Homo sapiens]	-1.44	-1.96
17	multifunctional protein ADE2 isoform 2 [Homo sapiens]	-1.04	-1.64
18	vimentin [Homo sapiens]	-2.78	-3.60
19	vimentin [Homo sapiens]	-4.04	-5.85
20	isocitrate dehydrogenase [NAD] subunit alpha, mitochondrial precursor [Homo sapiens]	1.17	1.53
21	transaldolase [Homo sapiens]	-1.06	-1.52
22	Lasp-1 protein	2.13	1.20
23	apolipoprotein L2 [Homo sapiens]	1.48	2.92
24	annexin A1 [Homo sapiens]	1.79	1.38
25	calponin-2 isoform a [Homo sapiens]	1.14	2.08
26	electron transfer flavoprotein subunit alpha, mitochondrial isoform a [Homo sapiens]	1.21	1.98
27	beta tropomyosin [Homo sapiens]	1.19	1.50
28	tropomyosin isoform [Homo sapiens]	1.89	2.43
29	vimentin [Homo sapiens]	-1.14	-1.97
30	ATP synthase subunit d, mitochondrial isoform b [Homo sapiens]	1.00	1.87
31	Ferritin, light polypeptide [Homo sapiens]	1.15	3.61
32	endoplasmic reticulum resident protein 29 isoform 1 precursor [Homo sapiens]	-1.55	-1.35
33	triosephosphate isomerase isoform 1 [Homo sapiens]	-3.46	-2.12
34	phosphoglycerate mutase 1 (brain) variant [Homo sapiens]	1.85	1.07
35	triosephosphate isomerase isoform 1 [Homo sapiens]	-6.25	-2.43
36	manganese-containing superoxide dismutase [Homo sapiens]	1.43	1.80
37	protein canopy homolog 2 isoform 1 precursor [Homo sapiens]	2.46	2.34
38	superoxide dismutase [Cu-Zn] [Homo sapiens]	1.41	1.58
39	stathmin 1 [Homo sapiens]	-1.09	-1.67
40	protein S100-A16 [Homo sapiens]	1.98	2.05
41	HIST2H4B protein [Homo sapiens]	1.02	2.11
42	mitogen-activated protein kinase scaffold protein 1 [Homo sapiens]	-10.89	-2.03
43	cytochrome C oxidase subunit 6B1 [Homo sapiens]	1.63	1.86

Figure S2
Li et al.

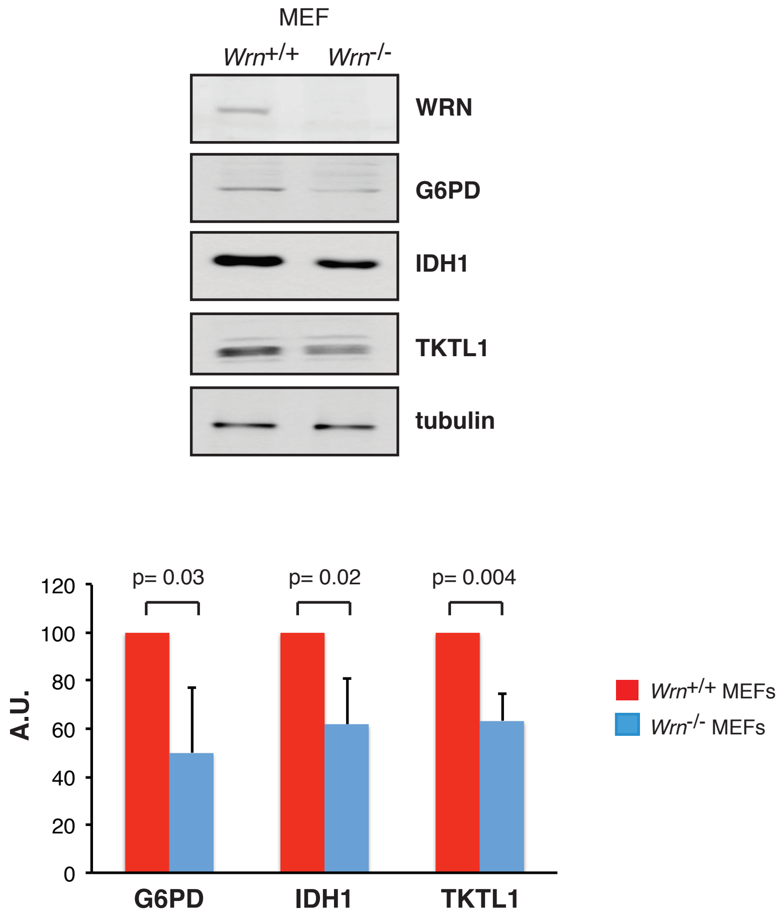

Figure S3
Li et al.

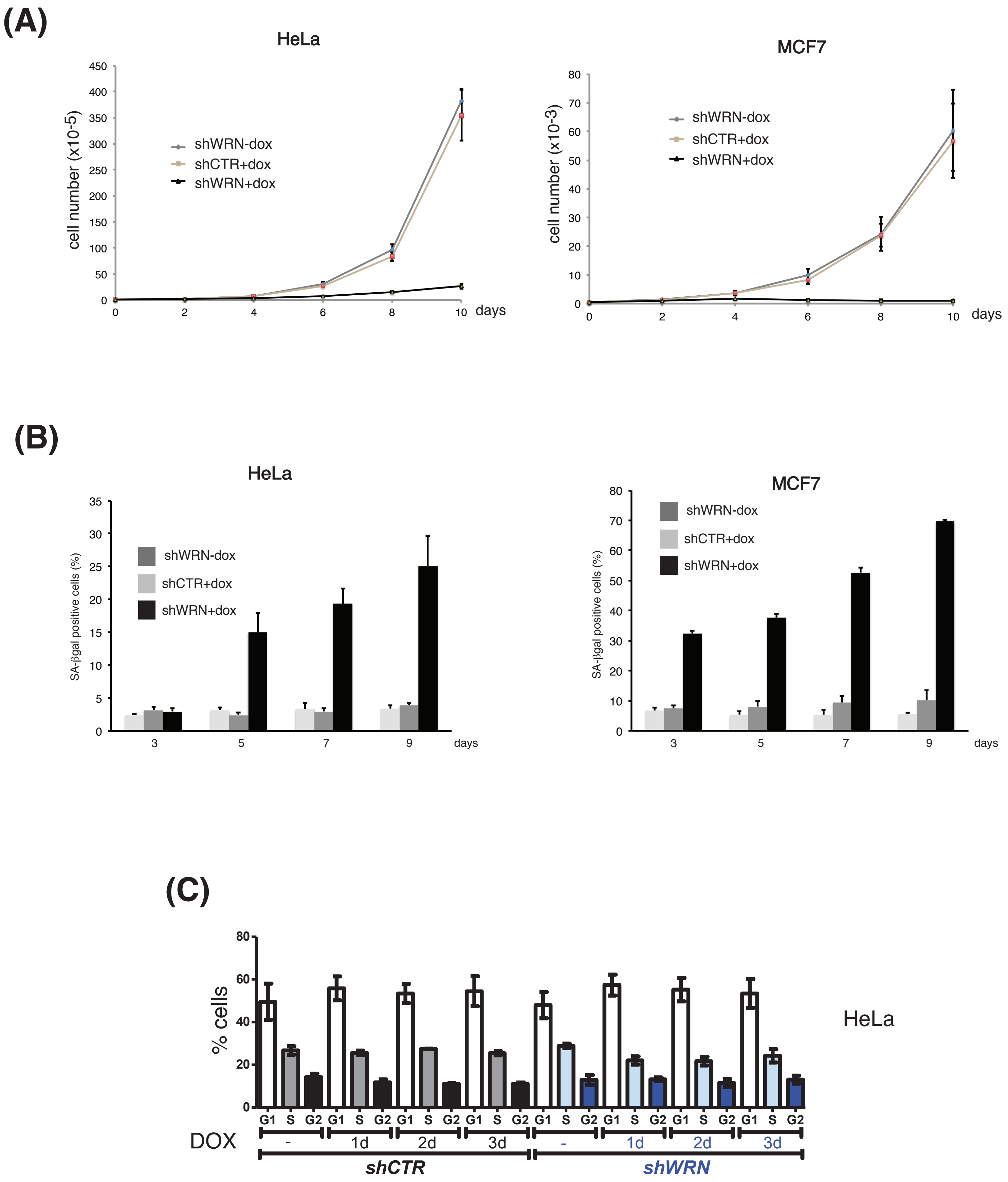

Figure S4
Li et al.

(A)

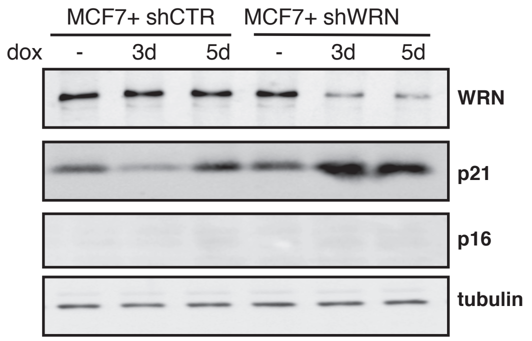

(B)

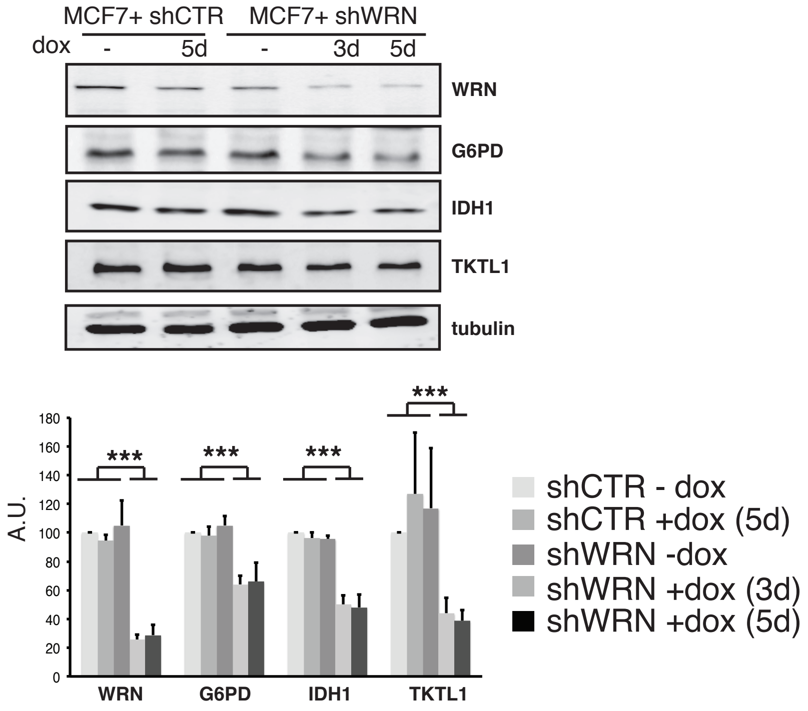

Figure S5
Li et al.

(A)

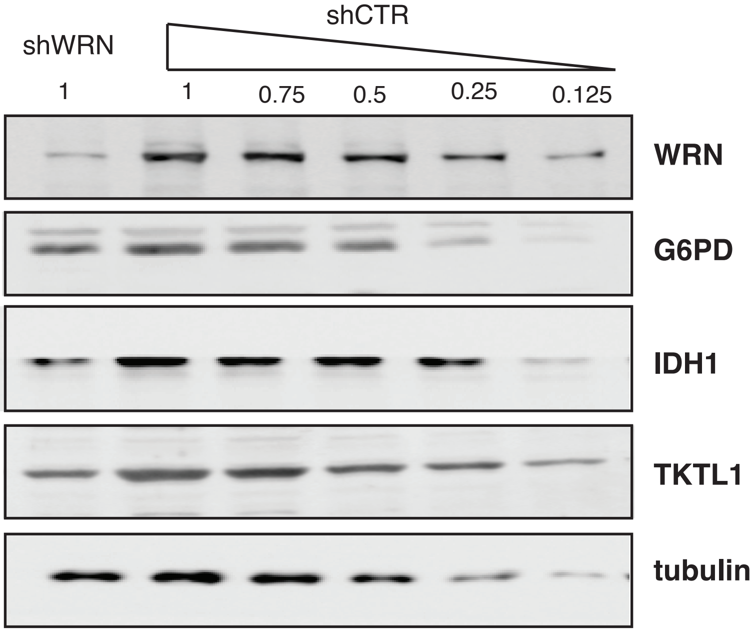

(B)

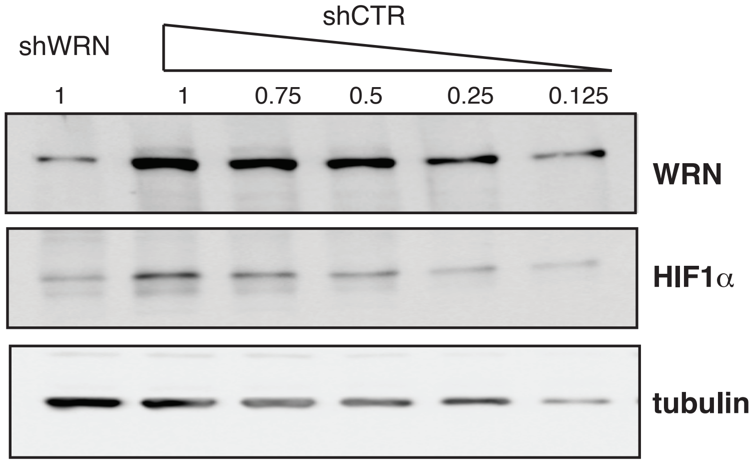

Figure S6
Li et al.

(A)

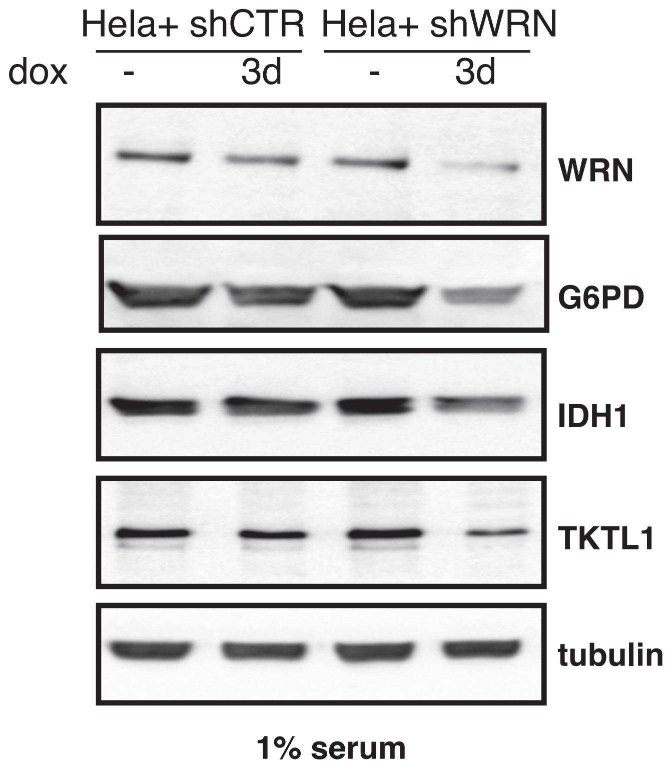

(B)

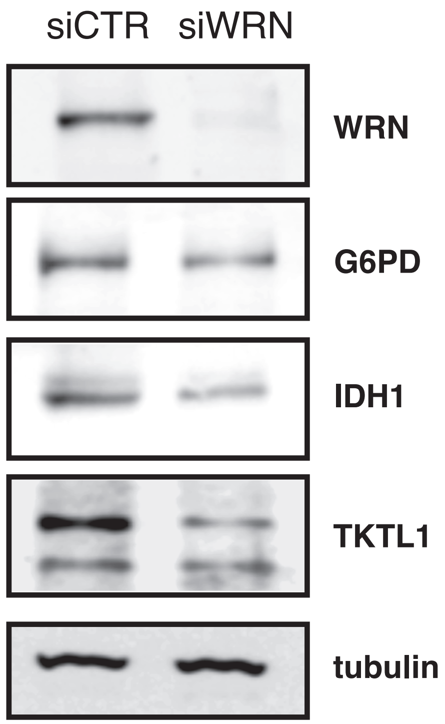

	siSCR	siWRN
**WRN**	100	6
**G6PD**	100	78
**IDH1**	100	43
**TKTL1**	100	31

Figure S7
Li et al.

(A)

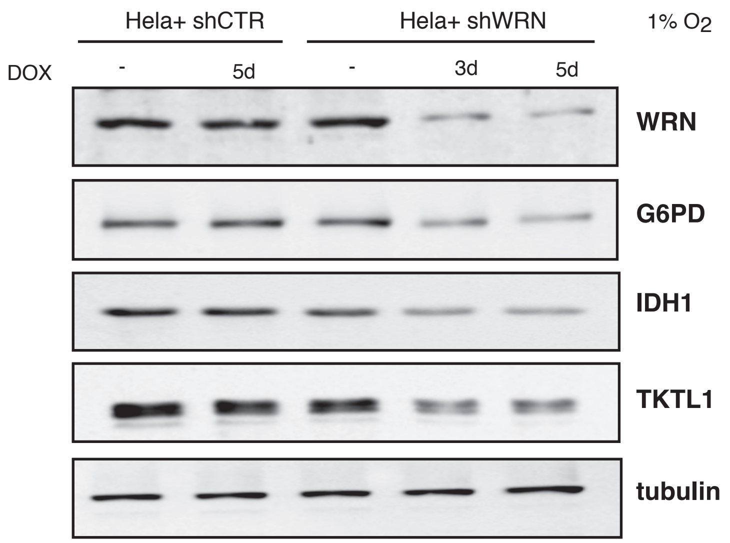

(B)

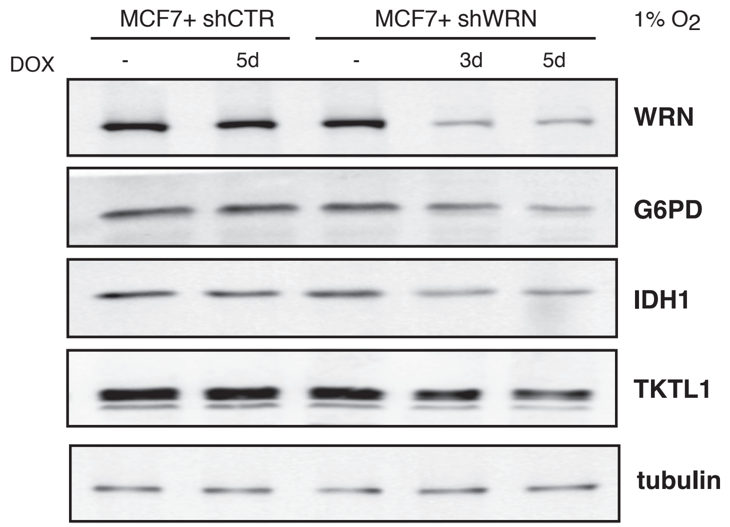

(C)

	shCTR		shWRN			
DOX	-	5d	-	3d	5d	
**WRN**	100	95	112	26	18	Hela
**G6PD**	100	108	96	53	47	Hela
**IDH1**	100	94	90	48	52	Hela
**TKTL1**	100	92	87	38	35	Hela

	shCTR		shWRN			
DOX	-	5d	-	3d	5d	
**WRN**	100	92	102	26	22	MCF7
**G6PDH**	100	96	92	65	34	MCF7
**IDH1**	100	94	96	53	44	MCF7
**TKTL1**	100	103	95	72	47	MCF7

Figure S8
Li et al.

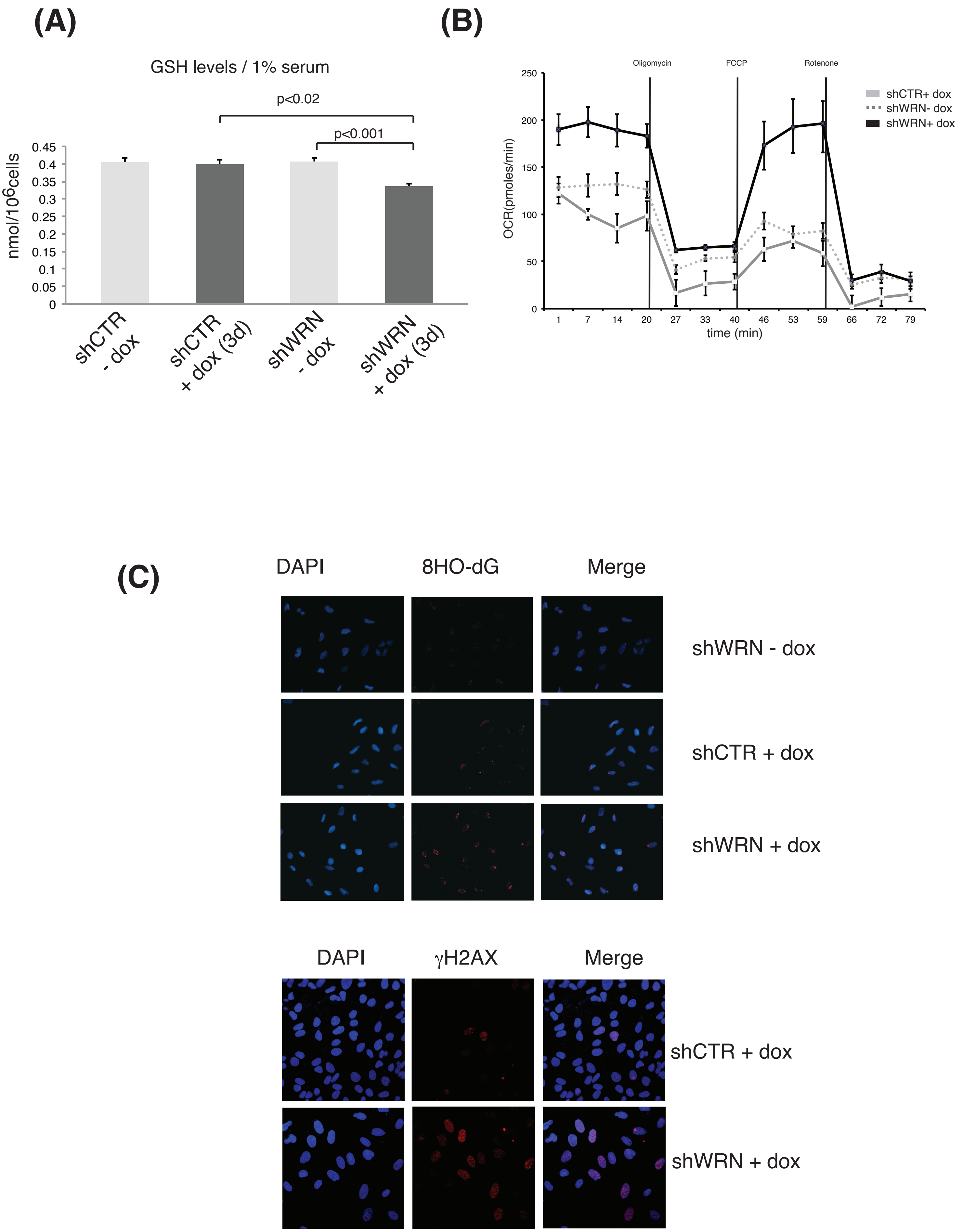

Figure S9
Li et al.

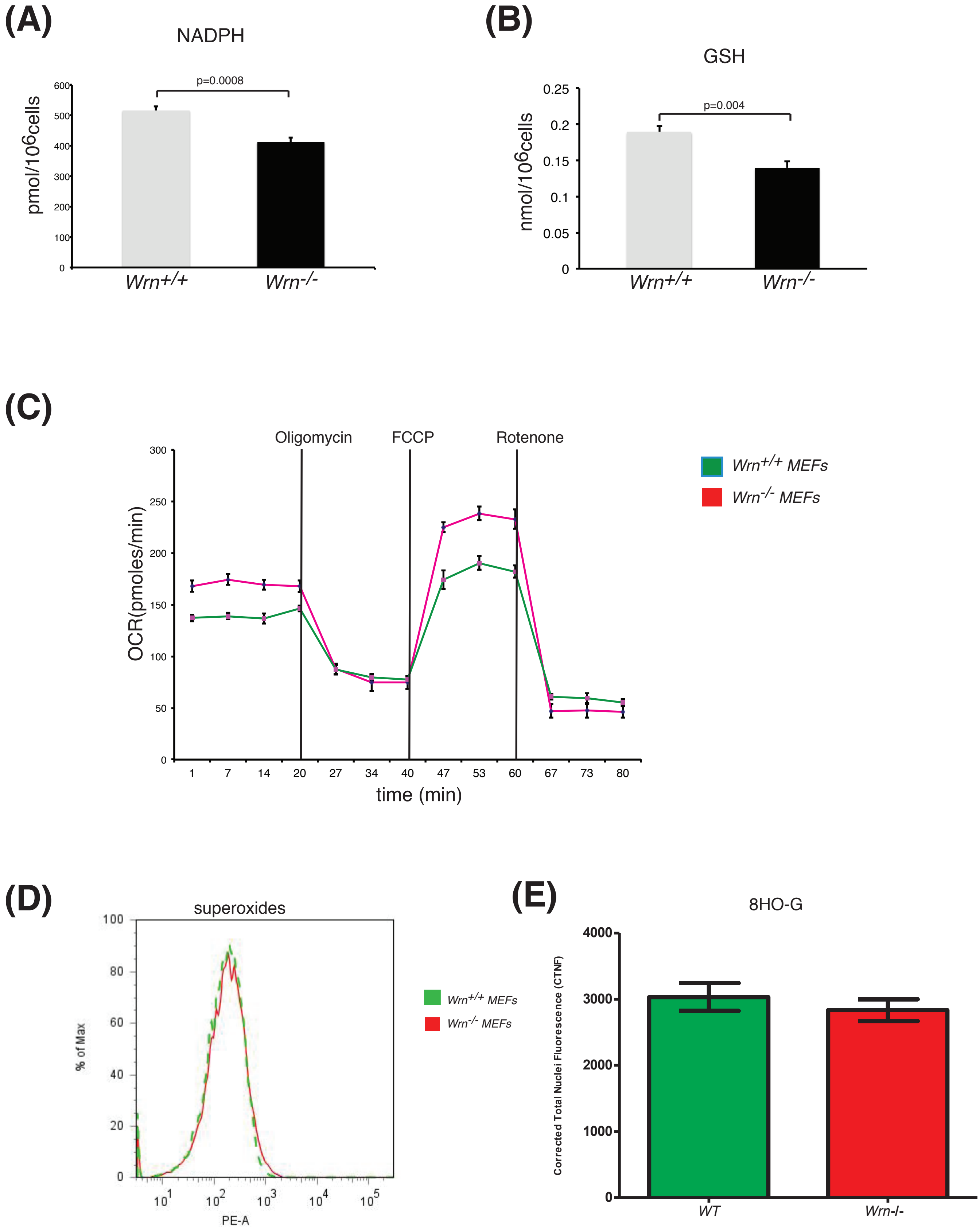

Figure S10
Li et al.

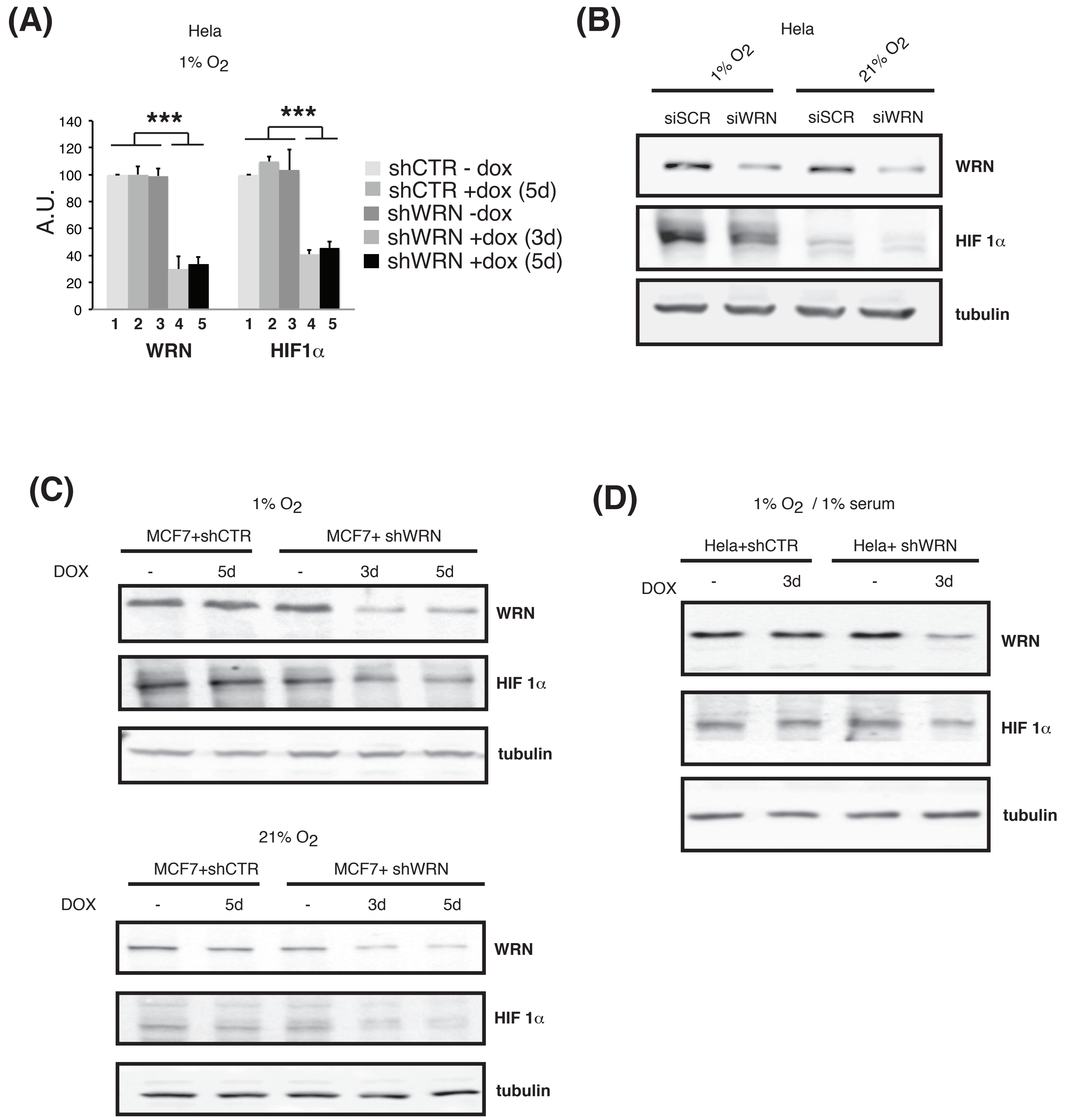

Figure S11
Li et al.

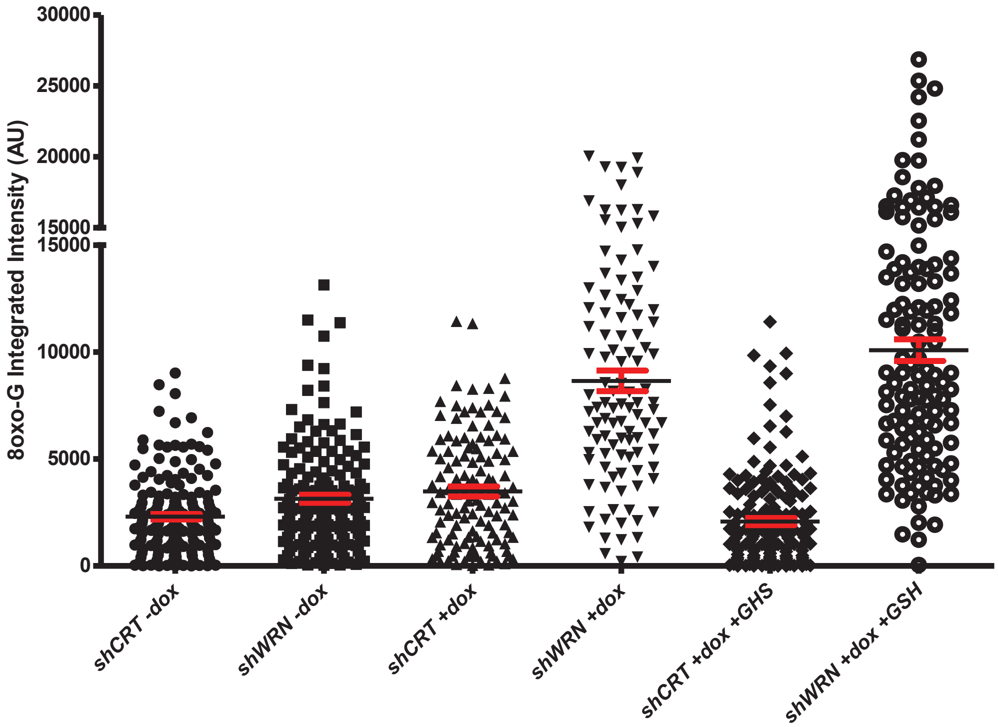

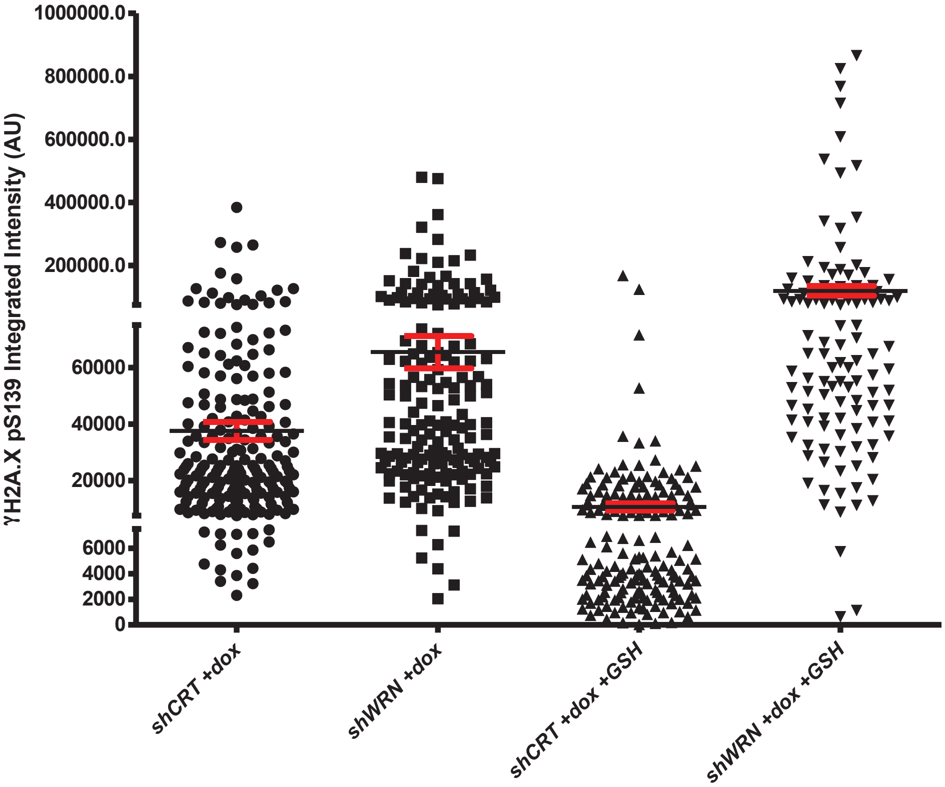

Figure S12
Li et al.

Supplementary Experimental Procedures

Transfections of siRNAs

On-target pool siRNA for human WRN (L-010378-00) and non-targeting pool siRNA (D-001810-10-05) were purchased from Dharmacon. For transfections, Hela cells were seeded in 60 mm plate and grown for 24 hours. The cells were transfected with 15 μl of 5uM WRN-targeting pool siRNA or non-targeting pool siRNA and 15 μl of the DharmaFECT 1 (T-2001-03; ThermoFisher Scientific) according to the manufacturer's protocol. Three days after transfection, cells were collected, washed with ice cold PBS and lysed in RIPA (150 mM NaCl, 1.0% NP40, 0.5% sodium deoxycholate, 0.1% SDS, and 50 mM Tris, pH 8.0) buffer. Protein levels were assessed with Western Blot using the appropriate antibody.

2-D DIGE analysis

AG14F/20 cells were infected with lentiviruses expressing shRNAs for WRN or GFP control and then selected with DMEM containing 400 μg/ml G418 and 200 μg/ml hygromycin for 7 days. DMEM with 1.0 μg/ml doxycycline (dox) was then added to the cell culture. At 3 and 5 day after dox induction, the cells were harvested and frozen at -80°C. The frozen cell pellets were shipped to Applied Biomics Inc. (Hayward, CA) where the 2-D DIGE analysis was carried out as follows. *Preparation of samples:* 200 μl of 2-D cell lysis buffer (30 mM Tris-HCl, pH 8.8, containing 7 M urea, 2 M thiourea and 4% CHAPS) were added to cell pellets. Protein concentration was measured using Bio-Rad protein assay method. *CyDye labeling:* For each sample, 30 μg of protein were mixed with 1.0 μl of diluted CyDye and kept in the dark on ice for 30 min. The labeling reaction was stopped by adding 1.0 μl of 10 mM lysine to each sample and incubating in the dark

on ice for an additional 15 min. The labeled samples were then mixed together. 2X 2-D Sample buffer (8 M urea, 4% CHAPS, 20 mg/ml DTT, 2% pharmalytes and trace amount of bromophenol blue), 100 ul destreak solution and Rehydration buffer (7 M urea, 2 M thiourea, 4% CHAPS, 20 mg/ml DTT, 1% pharmalytes and trace amount of bromophenol blue) were added to the labeling mix to bring the total volume to 250 μl. The samples were mixed well and centrifuged before loading into the strip holder. *IEF and SDS-PAGE:* After loading the labeled samples, IEF (pH3-10 Linear) was run following the protocol provided by GE Healthcare. Upon finishing the IEF, the IPG strips were incubated in freshly made equilibration buffer-1 (50 mM Tris-HCl, pH 8.8, containing 6 M urea, 30% glycerol, 2% SDS, trace amount of bromophenol blue and 10 mg/ml DTT) for 15 minutes with gentle shaking. Then the strips were rinsed in freshly made equilibration buffer-2 (50 mM Tris-HCl, pH 8.8, containing 6 M urea, 30% glycerol, 2% SDS, trace amount of bromophenol blue and 45 mg/ml iodoacetamide) for 10 minutes with gentle shaking. Next the IPG strips were rinsed in the SDS-gel running buffer before transferring onto 12% SDS-gels. The SDS-gels were run at 15 ^{o}C until the dye front ran out of the gels. *Image scan and data analysis:* Gel images were scanned immediately following the SDS-PAGE using Typhoon TRIO (GE Healthcare). The scanned images were then analyzed by Image Quant software (version 6.0, GE Healthcare), followed by in-gel analysis using DeCyder software version 6.5 (GE Healthcare). The fold change of the protein expression levels was obtained from in-gel DeCyder analysis.

Protein identification by Mass Spectrometry

Spot picking and Trypsin digestion: The spots of interest were picked up by Ettan Spot Picker (GE Healthcare) based on the in-gel analysis and spot picking design by DeCyder software. The gel spots were washed a few times then digested in-gel with modified porcine trypsin protease (Promega). The digested tryptic peptides were desalted using a

Zip-tip C18 (Millipore). Peptides were eluted from the Zip-tip with 0.5 μl of matrix solution (α-cyano-4-hydroxycinnamic acid (5 mg/ml in 50% acetonitrile, 0.1% trifluoroacetic acid, 25 mM ammonium bicarbonate) and spotted on the AB SCIEX MALDI plate (Opti-TOF™ 384 Well Insert). *Mass Spectrometry:* MALDI-TOF MS and TOF/TOF tandem MS/MS were performed on AB SCIEX TOF/TOF™ 5800 System (AB SCIEX). MALDI-TOF mass spectra were acquired in reflectron positive ion mode, averaging 4000 laser shots per spectrum. TOF/TOF tandem MS fragmentation spectra were acquired for each sample, averaging 4000 laser shots per fragmentation spectrum on each of the 7-10 most abundant ions present in each sample (excluding trypsin autolytic peptides and other known background ions). *Database search:* Both of the resulting peptide mass and the associated fragmentation spectra were submitted to GPS Explorer workstation equipped with MASCOT search engine (Matrix Science) to search the database of National Center for Biotechnology Information non-redundant (NCBInr). Searches were performed without constraining protein molecular weight or isoelectric point, with variable carbamidomethylation of cysteine and oxidation of methionine residues, and with one missed cleavage also allowed in the search parameters. Candidates with either protein score C.I.% or Ion C.I.% greater than 95 were considered significant.

Determination of cellular NADPH levels

Hela cells that were cultured under 20% or 1% O_2 were trypsinized, resuspended in PBS and collected by centrifugation. The cell pellets were then lysed with NADPH buffer (NADPH quantification kit 347-100, BioVision) by freeze/thaw two cycles (20min on dry-ice, 10min at room temperature) and centrifuged to collect supernatant. For NADPH extraction, the supernatant was incubated at 60°C for 30min. NADPH levels were determined at a wavelength of 450 nm on a UV spectrophotometer (1420 multilabel

Counter; PerkinElmer). The cellular NADPH content in each sample was calculated using a standard curve generated in parallel experiments.

Determination of cellular GSH levels

To determine the cellular levels of reduced glutathione (GSH), Hela cells that were cultured under 20% or 1% O_2, trypsinized, resuspended in PBS and than collected by centrifugation. Cells were then washed with ice-cold PBS, lysed with ice-cold glutathione buffer (ApoGSH: Glutathione Colorimetric Detection Kit 261-100, Biovision) for 10min on ice. 5% Sulfosalicylic acid was added to lysates, mixed well, and the supernatant was collected by centrifugation. Total GSH in the supernatant was determined by measuring absorbance at a wavelength of 405 nm using a UV spectrophotometer.

Transmission electron microscopy

For electron microscopy, cells were fixed (2% formaldehyde /2.5% glutarataldehyde in 0.1M Sodium cacodylate buffer, pH 7.4) at 4^oC overnight. Cells were processed at the USC Keck School of Medicine EM Facility and visualized on a JEOL JSM electron microscope. 10 randomly taken longitudinal sections of cells at 2500x magnification were used for quantification of mitochondrial density.

Mitochondrial oxygen consumption measurements

Cell were cultured on Seahorse XF-24 plates at a density of 5×10^4 cells/well. On the day of metabolic flux analysis, cells were changed to unbuffered DMEM (DMEM base medium supplemented with 25 mM glucose, 1 mM sodium pyruvate, 31 mM NaCl, 2 mM GlutaMax, pH 7.4) and incubated at 37 °C in a non-CO_2 incubator for 1 h. Oxygen consumption rate (OCR) for the cells was performed with Seahorse XF24 analyzer (Seahorse Bioscience, North Billerica, MA). The data are representative of three

independent experiments. In each experiment, each data point is the average of five independent measurements.

Determination of mitochondrial membrane potential

For measurement of Mitochondria Membrane Potential (MMP), 10^6 cells were incubated with 1μg/ml JC-1 (5,5',6,6'-tetrachloro-1,1',3,3'-tetrahetylbenzimidazolylcarbocyanide iodide; Immunochemistry MitoPt JC-1, cat#924) for 20 min in the dark. The cells were then washed 3 times and samples were analyzed by Flow Cytometry (FACSCanto II, BD Biosciences). Monomeric JC-1 (low MMP) is visualized at 527 nm (green fluorescence) while JC-1 aggregates (high MMP) are visualized at 590 nm (red fluorescence). Uncoupling with carbonyl cyanide 3-chlorophenylhydrazone (CCCP) (50μM for 1 hour) was used as positive control for membrane depolarization.

Immunofluorescence microscopy

2.5 to 3.5×10^5 cells were seeded in an 8 well chamber slide (Lab-Tek) and incubated at 37^0 overnight. The cells were then washed twice with PBS and fixed with cold methanol at -20°C for at least 20 minutes, blocked with 5%BSA in PBS supplemented with 5% Normal Donkey Serum (NDS) for an hour at RT and incubated overnight at 4°C with 8oxoG DNA Lesion antibody (SC-130914), or Histone H2A.XS139phosph (39117; Active Motif) or Normal Mouse IgM (SC-3881) at dilution 1/500 in the blocking buffer. After extensively washing with PBS, the cells were incubated with Alexa Fluor 594 donkey or rabbit anti-mouse antibody (1;200 dilution, Molecular Probes A21203) in 5% BSA for an hour, washed extensively and mounted with Vectashield mounting media containing DAPI (VECTOR H-1200). Cells were analyzed by a confocal microscopy with 60X

magnification oil immersion objective. The images were processed using ImageJ or Metamorph.

Detection of superoxide radical anions

For superoxide measurements, the cells were washed with PBS, then incubated with PBS containing 3.0 µM MitoSox (M36008; Invitrogen) at 37°C for 10min. The dye was then removed and the cells were washed with PBS, The cells were observed with fluorescent microscope. MitoSox treated-cells were trypsinized, neutralized with DMED containing 10% FBS, centrifuged and fixed with 2% paraformaldehyde. The cells were then resuspended in PBS containing 2% FBS and analyzed by flow cytometry (FACSCanto II, BD Biosciences).

Detection of peroxides

MEF and Hela cells carrying the constructs shGFP and shWRN were growth in petri dishes in the presence or absence of doxycycline (dox) in complete DMEM for the indicated periods of time. Cells were then trypsinized and washed once with completed DMEM. The cell pellet was resuspended in DPBS (containing chloride and magnesium), and incubated with PF6-AM (5µM final concentration) or Mitochondrial Peroxy Yellow 1 (mitoPY1; 5µM) for 1h at 37°C in 1% CO_2. The cells were washed once with DPBS supplemented with 1% FCS and analyzed by flow cytometry (FACSCanto II, BD Biosciences). The data were processed using FlowJo software.

Analysis of mRNAs by reverse transcriptase quantitative PCR

Cells were growth in 1% or 20% O_2 and three days after the addition of doxycycline they were trypsinized, resuspended in ice cold PBS and counted. For RNA extraction, 1 x 10^6 cells were lysed using TRIZOL (15596-026; Invitrogen) following the manufacturer

instructions. RNA solution was treated with DNase (TURBO DNA-*free*, AM-1907; Ambion) and the same volumes of RNAs were subjected to first strand cDNA synthesis using Maxima H Minus kit (K1651; Thermo Scientific). For qPCR we used the following set of primers: TUBA-1F 5'-ATGCGTGAGTGCATCTCCATC, TUBA-128R 5'-GCCAAGTGACAAGACCATTGG; WRN-4665F 5'-CAAGCGGTGAAAGCTGGCTGC, WRN-4768R 5'-GAGTTGACGGGAGGGTTTCGG; HIF1α-1825F 5'-AGCAGTTCCGCAAGCCCTGA, HIF1α-1931R 5'-GCCACCACTACCACTGCCAC. Quantitative PCR assays were carried out using C1000 Thermal Cycler using CFX96 System (BioRad).
